# Genomic Surveillance of Epiphytic *Pseudomonas syringae* Highlights Shared Reservoirs and Cross‐Habitat Threats to Cherry Orchards and Nearby Woodland Plants

**DOI:** 10.1111/mpp.70208

**Published:** 2026-02-16

**Authors:** Ziyue Zeng, John W. Mansfield, Andrea Vadillo‐Dieguez, John Connell, James Irvine, Michelle T. Hulin, Fernando Duarte Frutos, Mojgan Rabiey, Nastasiya F. Grinberg, Richard J. Harrison, Xiangming Xu, Robert W. Jackson

**Affiliations:** ^1^ Niab Cambridge UK; ^2^ School of Biosciences and Birmingham Institute of Forest Research University of Birmingham Birmingham UK; ^3^ Faculty of Natural Sciences Imperial College London UK; ^4^ Department of Plant Soil and Microbial Sciences Michigan State University East Lansing Michigan USA; ^5^ Niab at East Malling Kent UK

**Keywords:** bacterial canker, epidemiological surveillance, epiphyte, pathogen reservoirs, *Prunus*, *Pseudomonas syringae*

## Abstract

Plant surfaces host diverse microbial communities acting as reservoirs for pathogenic lineages, yet the ecological dynamics and evolutionary consequences of such reservoirs remain underexplored. We conducted landscape‐scale genomic surveillance of *Pseudomonas syringae* on symptomless leaves of cultivated cherry in orchards and wild plant species in adjacent woodlands across the UK, aiming to understand how phyllosphere populations contribute to the emergence of bacterial canker. Whole genome sequencing of 540 isolates collected over two years and across four regions revealed 10 diverse *P. syringae* phylogroups (PGs) on symptomless leaves. Both orchard and woodland environments harboured a similar range of PGs, but recovery frequency was very different. PG2d strains dominated cherry orchards, whereas PGs 2b and 13a were prevalent in woodlands. Certain PG2d subclades, recovered from both environments, caused disease on cultivated and wild cherry leaves. Additional strains were found to be pathogenic to *Phaseolus* bean pods. The pathogens of cherry were characterised by the presence of genes encoding the synthesis of the pathotoxin syringolin A and a subset of effector proteins including HopAW1, AvrRpm1 and HopAR1. Resolution of subclades within PG2d provided insights into the emergence of virulent epiphytic strains that have not yet reached the mostly northerly sampling sites but are threats to both cultivated and environmental *Prunus* spp. Fine‐scale analysis of subclade PG2d‐3 revealed potential divergence between orchard and woodland populations, with 49 genes exclusive to a woodland lineage. Thirty‐eight of these genes were found within prophages, indicating the potential role of bacteriophage‐mediated horizontal gene transfer in adaptation to non‐agricultural reservoirs.

## Graphical abstract

Highly virulent epiphytic *Pseudomonas syringae* PG2d strains are widespread in UK cherry orchards and woodlands, varying in abundance and threatening domestic and wild cherry health.

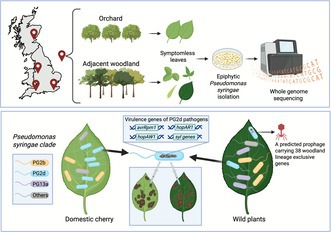

## 1 Introduction

Extensive studies have focused on the direct interactions between pathogens and their host plants (Ali and Alsayeqh 2022; Mansfield et al. 2012). However, outbreaks of plant and human diseases have been increasingly linked to the survival of pathogens away from their hosts in reservoirs of potential infection (Donati et al. 2020; Monteil et al. 2016; Morris et al. 2013). These overlooked reservoirs, particularly wild environments, may play a critical role in pathogen persistence, diversification and dispersal (McCann 2020; Monteil et al. 2013; Morris et al. 2013). The concept of epidemiological surveillance, whereby high‐throughput whole genome sequencing (WGS) is used to characterise pathogens in the wider environment, has been proposed as an approach to warn against outbreaks of disease caused by emerging strains of pathogens, both in the field of human health (Deng et al. 2016) and more recently plant disease (Abrahamian et al. 2019; Hemara et al. 2025; McCann 2020; Nizamani et al. 2023; Straub et al. 2021; Weisberg et al. 2021).

Bacteria recognised as members of the *Pseudomonas syringae* species complex cause diseases on a wide range of crops (Mansfield et al. 2012; Yang et al. 2023). *P. syringae* has been differentiated as a collection of 13 phylogroups (PGs) by Berge et al. (2014) using multilocus sequence typing (MLST) on 216 strains and further refined by a study of 139 sequenced strains into 19 genomospecies, based on 95% average nucleotide identity (ANI) by Gomila et al. (2017). Within individual phylogroups there are pathogens of different plant hosts. A good example is PG2, which is reported to contain strains causing economically important disease on plants as diverse as wheat, pea, and apple caused by *P. syringae* pvs. *atrofaciens* (Butsenko et al. 2021), *pisi* (Martín‐Sanz et al. 2012), and *papulans* (Kerkoud et al. 2000), respectively. Bacterial canker of cherry and other *Prunus*, reviewed by Hulin et al. (2020), is known to be caused by members of PGs 1, 2, 3 and 7, with major outbreaks of the disease attributed to *P. syringae* pv. *morsprunorum* (Psm) races 1 (Psm1 in PG3) and 2 (Psm2 in PG1) and *P. syringae* pv. *syringae* (Pss in PG2). As a crucial constraint on cherry production in many countries, bacterial canker represents a global issue for orchard management, where severe outbreaks can result in substantial tree loss, reduced fruit yield and long‐term economic impact (Marroni et al. 2024; Vadillo‐Dieguez et al. 2025).

Despite their taxonomic divergence, the strains of *P. syringae* attacking cherry have been found to contain common groups of effector proteins that are delivered into plant cells through the type III secretion system (T3SS), and also to synthesise low molecular weight phytotoxins, notably syringomycin and syringolin A (Dudnik and Dudler 2014; Scholz‐Schroeder et al. 2001; Vadillo‐Dieguez et al. 2024). Genetic dissection has confirmed the roles of effectors and toxins in the virulence to cherry tissues of PG2 strain Pss 9644 (Vadillo‐Dieguez et al. 2024). The relative importance of effectors and toxins to pathogenicity of *P. syringae* varies between PGs, with PG2 strains having fewer effector encoding genes but secreting more toxins than those in, for example, PGs 1 or 3 (Newberry et al. 2019; Vadillo‐Dieguez et al. 2024; Xin et al. 2018). The complex population structure and virulence mechanisms make the *P. syringae*–cherry interaction a valuable system for exploring how pathogenic bacteria evolve, adapt and spread.

Although the concept of reservoirs of infection typically relates to bacteria away from potential hosts, in crop plants a significant reservoir has been recognised on plant surfaces (Lindow and Brandl 2003; Morris et al. 2013; Vorholt 2012). *P. syringae* spp. are common components of epiphytic microbiomes and they multiply within the plant surface ecosystem without causing disease symptoms or reducing plant growth (Crosse 1966; Hirano and Upper 2000; Hulin et al. 2020). In an earlier study we carried out a ‘snapshot’ survey of the presence of epiphytic *P. syringae* in the phyllosphere of cultivated cherry using one sampling time in orchards across England (Hulin et al. 2023). We sequenced 166 epiphytic strains and found considerable phylogenetic diversity occurring on the plant surface. The potential for exchange of virulence factors by horizontal gene transfer (HGT) occurring within the dynamic bacterial communities on the plant surface has long been postulated (Sawada et al. 1999). This has now been confirmed through the demonstration that bacteriophage (phage)‐mediated horizontal transfer of a gene encoding the type III secreted effector (T3E) protein HopAR1 occurs within the active phyllosphere environment on the cherry leaf (Hulin et al. 2023). These findings highlight the importance of the phyllosphere as an active ecological niche where virulence genes can circulate, potentially giving rise to novel pathogenic lineages even in the absence of disease symptoms.

Reservoirs of infection close to cultivated plants can be particularly important for perennial crops (McCann 2020). For example, fruit trees may be grown in orchards adjacent to woodlands that contain related plant species potentially harbouring pathogenic strains. In contrast to the clonally propagated sweet cherry cultivated in orchards, wild cherry populations exhibit greater genetic variation in resistance to *P. syringae* infection (Hulin et al. 2022). A similar pattern has been observed in kiwifruit, where wild‐derived *Actinidia* germplasm and commercial varieties show differential resistance to *P. syringae* pv. *actinidiae* (Su et al. 2024; Wang et al. 2020). Broader genomic analyses highlight substantial natural diversity within kiwifruit germplasm (Nazir et al. 2024), reinforcing the importance of host genetic variation in shaping disease resilience in perennial crops. As such, genetically diverse wild hosts can maintain pathogen populations over long periods and may carry strains not present in managed crops, but they may serve as important reservoirs of new outbreaks of crop disease. To compare *P. syringae* populations between managed and wild environments, we carried out surveillance of strains of *P. syringae* in commercial cherry orchards and wild cherry as well as other related plant species in nearby woodlands. Pathogenicity tests on cultivated and wild cherry and the unrelated *Phaseolus vulgaris* (French bean) were carried out on 203 epiphytic strains recovered in 2021. We have designed experiments to test the following hypotheses:

1. Cherry trees in orchards and related wild plants in nearby woodlands harbour different populations of epiphytic *P. syringae*.
2. Bacteria pathogenic to crops are present as epiphytes on wild plants.
3. Populations of *P. syringae* in the phyllosphere vary significantly in different regions.
4. Highly virulent *P. syringae* strains survive on the plant surfaces throughout the UK without causing canker symptoms.

In addition to addressing these questions, our genomic surveillance approach has allowed the identification of the widespread emergence of highly virulent PG2d strains that threaten both cultivated and wild cherry species.

## 2 Results

### 2.1 The Diversity of Epiphytic *P. syringae* Phylogroups and Clades in Orchards and Woodlands

**FIGURE 1:**
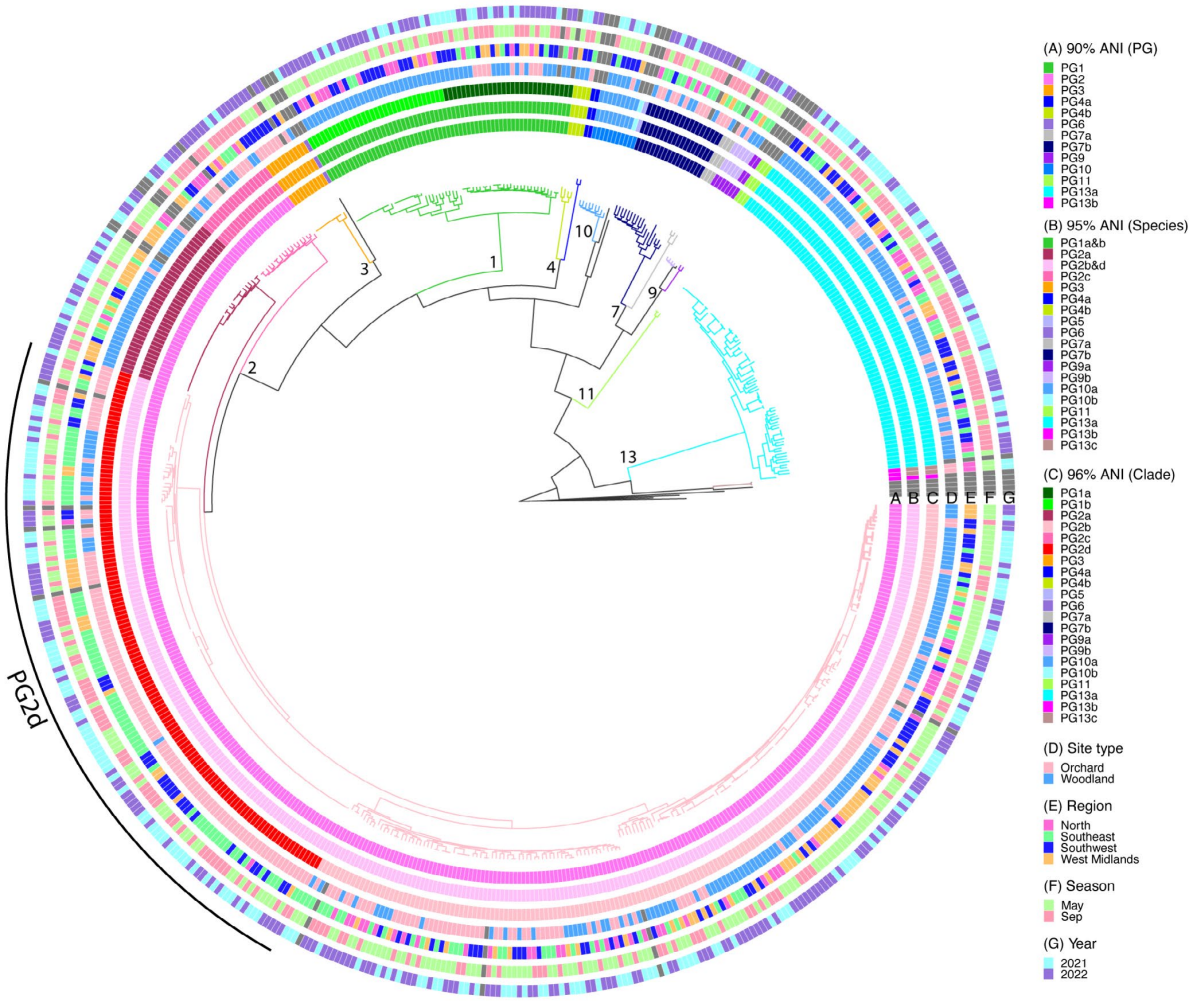
Phylogroups (PGs) of epiphytic *Pseudomonas syringae* strains recovered from regional samplings of orchards and woodlands in 2021 and 2022. A maximum‐likelihood core genome phylogeny tree of 540 *P. syringae* strains isolated from samplings and 39 reference strains. Rings A, B and C show classifications of PG, species and clade based on average nucleotide identities (ANIs) of 90%, 95% and 96%, respectively, with the non‐ *P. syringae* pseudomonad reference strains marked in grey. Rings D–G show information on strain isolation. Reference strains downloaded from GenBank are marked in dark grey in rings D–G. Numbers near branches show PG classification. Colours of tree branches show classification of species.

To survey epiphytic populations of *P. syringae* in orchard and non‐agricultural environments, repeated seasonal samplings of leaf surfaces were performed in May and September in 2021 and 2022, and in four regions in the UK: the Southeast (SE), Southwest (SW), West Midlands (WM) and North (N, Scotland). Within each region, three cherry cultivars in each orchard and six plant species in each adjacent woodland were sampled. In total, over 7000 environmental isolates were recovered and screened via PCR to identify *P. syringae*. A subset of 1101 strains was selected for WGS (Table S1). The sequences of 540 strains shared at least 95% ANI with a known *P. syringae* sequence in GenBank (Benson et al. 2018). The diversity of strains recovered from leaf surfaces is illustrated in Table 1 and Figure 1. Members of PGs 1, 2, 3, 4, 7, 9, 10 and 13 were identified with 90% ANI. Species were further discriminated at 95% ANI. Overall, the more common species recovered were PG2b&d (57.6%), PG13a (14.1%), PG1a&b (10.7%) and PG2a (5.9%) (Table 1). By raising the cut‐off of ANI to 96%, the species PG1a&b and PG2b&d were further refined into distinct clades; PGs 1a and 1b, and 2b and 2d, respectively.

**TABLE 1:** Numbers and percentages of strains in different phylogroups (PGs), species and clades amongst the 540 *Pseudomonas syringae* strains sequenced from seasonal samplings. The 540 *P. syringae* strains recovered were members of 10 PGs and included 12 species and 14 clades based on 90%, 95% and 96% average nucleotide identities (ANIs). Results are marked in blue if they were the same as those at PG level (ANI = 90%).

Phylogroup (ANI 90%) and number of strains	Percentage of strains	Species (ANI 95%) and number of strains	Percentage of strains	Clades (ANI96%) and number of strains	Percentage of strains
PG1 (58)	10.7%	PG1a&b (58)	10.7%	PG1a (27)	5%
				PG1b (31)	5.7%
PG2 (361)	66.9%	PG2a (32)	5.9%	PG2a (32)	5.9%
		PG2b&d (311)	57.6%	PG2b (187)	34.6%
				PG2d (124)	23%
		PG2c (18)	3.3%	PG2c (18)	3.3%
PG3 (8)	1.5%	PG3 (8)	1.5%	PG3 (8)	1.5%
PG4b* (4)	0.7%	PG4b* (4)	0.7%	PG4b* (4)	0.7%
PG7a* (2)	0.4%	PG7a* (2)	0.4%	PG7a* (2)	0.4%
PG7b* (17)	3.1%	PG7b* (17)	3.1%	PG7b* (17)	3.1%
PG9 (5)	0.9%	PG9b* (5)	0.9%	PG9b* (5)	0.9%
PG10 (7)	1.3%	PG10a (7)	1.3%	PG10a (7)	1.3%
PG13a* (76)	14.1%	PG13a* (76)	14.1%	PG13a* (76)	14.1%
PG13b* (2)	0.4%	PG13c* (2)	0.4%	PG13c* (2)	0.4%

* Groupings defined from this study based on ANIs and different from reference strains, which are representatives of established clades.

**FIGURE 2:**
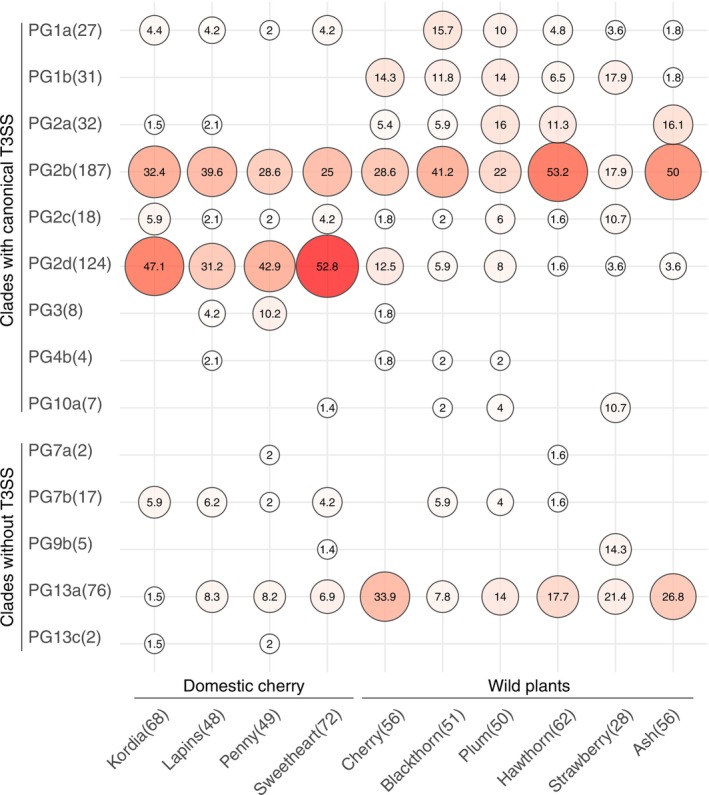
Percentages of *Pseudomonas syringae* strains of each clade (average nucleotide identity [ANI] 96%) isolated from different cherry cultivars in orchards or plant species in woodlands. The numbers in the circles show the percentages that each clade takes up from the total numbers of strains isolated from each cherry cultivar or wild plant species. The size of the circle and the intensity of its red colour are proportional to the number inside the circle. The total numbers of strains of each category are marked in parentheses, on the *x*‐axis next to the cultivar or wild plant name and the *y*‐axis next to the clade name. Wild plant species are listed (left to right) in order of their relationship to cherry, from closest to most distantly related. Clades with the canonical type III secretion system (T3SS) are grouped.

Amongst the woodland tree species, the percentages of members of different clades were overall closely comparable (Figure 2). The clade PG2b dominated almost all plant hosts, with the exception that PG13a was the most prevalent clade in wild cherry and wild strawberry. A clear outlier in the woodland data was from the ground covering plant, wild strawberry, from which far fewer PG2 stains were recovered. As the numbers of *P. syringae* isolates sequenced from wild strawberry were low, these numbers were combined for initial quantitative analysis with data from other species to present ‘Woodland’ isolates representing the reservoir of *P. syringae* in the natural environment close to orchards. This approach was supported by the finding that even hawthorn, wild strawberry and ash, being distantly related to *Prunus*, harboured epiphytes pathogenic to cherry (as shown below).

**FIGURE 3:**
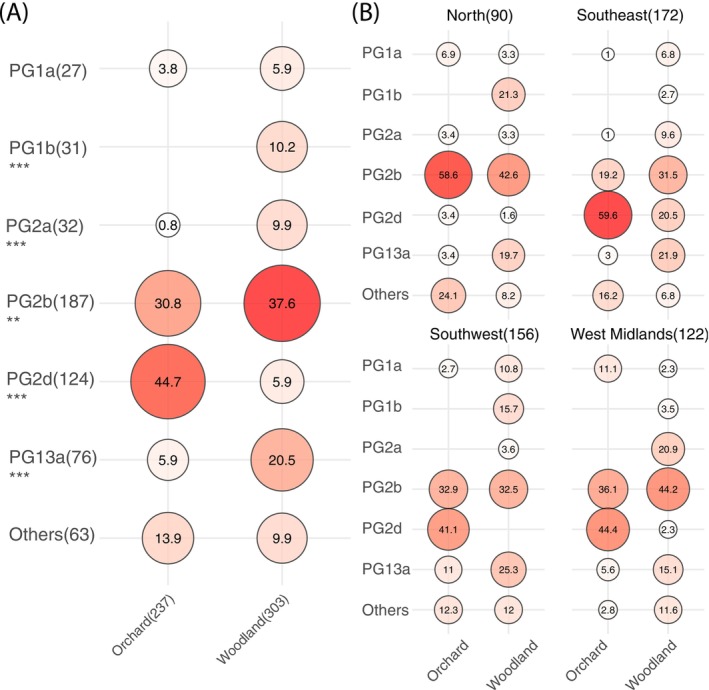
Percentages of strains of each clade of *Pseudomonas syringae* (96% average nucleotide identity [ANI]) isolated from orchards or woodlands in different regions. (A) All regions combined. (B) Individual regions. The numbers in the circles show the percentages that each clade takes up from the total numbers of strains isolated from orchards or woodlands in combined samplings or in each region. The size and intensity of the colour of circles visualise the percentages in each circle. Clades with small total numbers of strains were combined to be ‘Others’. Clades in which percentages differed significantly between sites are marked in (A) (Binomial test, **p ≤ 0.01, ***p ≤ 0.001). The total numbers of strains of each category are marked in brackets, on the *x*‐axis next to the site type name and the *y*‐axis next to the clade name in (A). The total numbers of strains isolated from each region are marked in parentheses next to the region name in (B).

Similarly, PG frequencies were closely comparable between cherry cultivars in the ‘Orchard’ samples (Figure 2). However, there were clear statistically significant differences comparing combined numbers from orchards and woodlands (237 and 303 strains sequenced, respectively, Figure 3A). Although PGs 2b and 2d together (the PG2b&2d species) were the predominant clades for both site types, occupying 75.5% and 43.5% of strains in orchards and woodlands, respectively, PG2b strains were more prevalent in woodlands and occupied the largest proportion (37.6%) of all the strains isolated from the natural environment. By contrast, PG2d was the most abundant clade in orchards (44.7%), followed by PG2b (30.8%). No strains of PG1b were recovered from cultivated cherry and most strains of PG13a, PG1a and PG2a were isolated from woodlands. The contrasts between the prevalent PGs 2b and 2d strains isolated from orchards and woodlands were consistent across seasons, years and the three regions sampled in England (Figures 3B and S1). Intriguingly, in contrast to the distinct abundance of PG2d in England, especially in the Southeastern orchards, PG2d was almost absent from sites in Scotland (North), the caveat being that overall, far fewer sequenced isolates from the most northerly site were confirmed to be *P. syringae* (Figure 3B and Table S2).

### 2.2 Pathogenicity of Isolates From 2021—Identification of Widespread Highly Virulent Subclades Within PG2d

The pathogenicity of all *P. syringae* strains (203) recovered in two samplings in 2021 was tested via leaf infiltration assays on cultivated cherry cv. Sweetheart and a subset was also tested on a wild cherry accession Howley Wood (Hulin et al. 2022). We confirmed that the level of necrosis observed in leaves after inoculation was directly correlated with bacterial populations (Figure S2). All strains were also tested on pods of French bean via stab inoculation.

**FIGURE 4:**
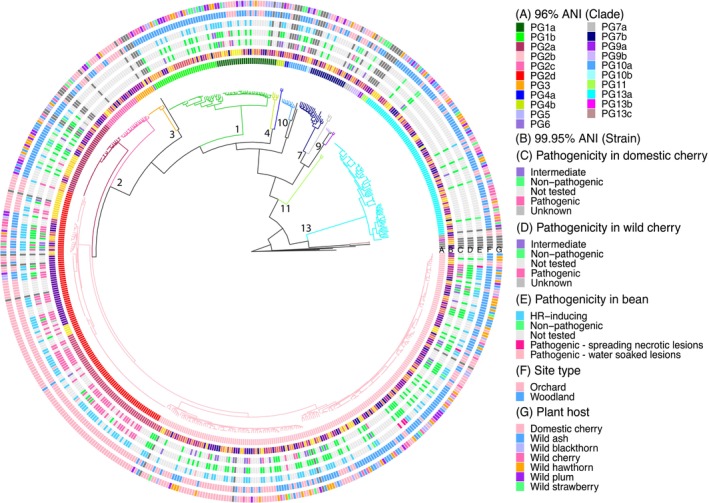
Pathogenicity of *Pseudomonas syringae* strains from the 2021 samplings linked to phylogroup, species, clade and PG2d subclade classification. The maximum‐likelihood phylogeny tree of 540 *P. syringae* strains was determined as in Figure 1. Rings A and B show classifications of clades and subclades based on average nucleotide identities (ANIs) of 96% and 99.95%, respectively. The colour code of tree branches shows classification of species (95% ANI). Rings C–E present data on pathogenicity to cultivated cherry cv. Sweetheart, wild cherry (a partially susceptible wild cherry accession, Howley Wood) and French bean. Pathogenic strains indicated the same level of virulence as the pathogen control whilst nonpathogenic strains did not show any disease symptom. Strains causing some but limited symptoms were categorised as intermediate. Strains giving inconsistent results were recorded as unknown pathogenicity. Each bacterial strain was infiltrated on at least three different leaves of each type of cherry. Symptom development was scored at 3, 6 and 9 days after inoculation.

The results of the pathogenicity tests are shown in combination with a phylogenetic tree using the 99.95% ANI comparison (Figure 4, ring B). Strains pathogenic to cultivated cherry were found from PGs 1a, 2a, 2b, 2d and 10a. By contrast, pathogens of wild cherry were mainly PG2d strains isolated from orchards (Figure 4, rings C & D and Figure S3). Reactions on bean were assessed to examine the presence of pathogens to a plant unrelated to any species of origin and also ability to trigger the hypersensitive reaction (HR). Nine strains, all from PGs 2b and 2d, were pathogenic to bean producing either spreading necrotic or water‐soaked lesions, whereas the vast majority triggered an HR in bean pods (Figure 4, ring E and Figure S4). Further breakdown of the frequency of pathogens of cherry isolated from different plants is given in Figure S3. Pathogens of cultivated cherry were recovered from each of the wild species, supporting our decision to group all woodland species together for comparative purposes.

To investigate the virulence factors underlying these pathogenicity patterns, we examined T3SS‐ and effector‐associated genes. Genes encoding known effectors were detected in all phylogroups (Figure S5). The canonical tripartite pathogenicity island (T‐PAI) T3SS was found only in some clades based on our screen (Figure S5); however, this does not exclude the presence of non‐canonical PAI types, such as the atypical pathogenicity island (A‐PAI) T3SS reported in PG13 (Dillon, Thakur, et al. 2019; Patterson et al. 2025).

**FIGURE 5:**
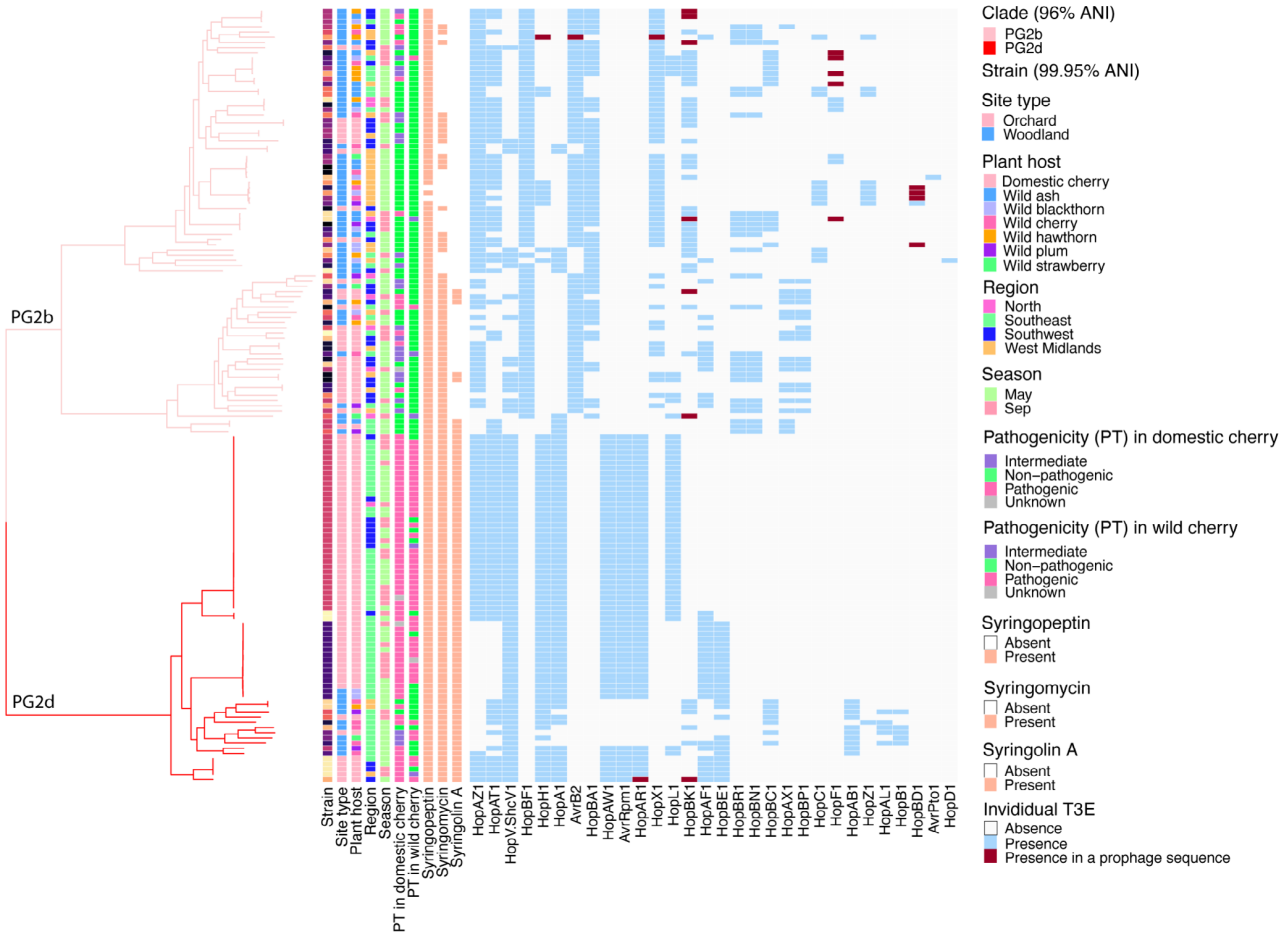
Presence of genes encoding type III effector (T3E) proteins and toxin biosynthesis in relation to the pathogenicity of *Pseudomonas syringae* strains recovered in 2021 within subclades of phylogroups 2b and 2d, and their site of origin. The dendrogram is a subtree extracted from the core genome phylogeny in Figure 1. Classification at the clade (average nucleotide identity [ANI] 96%) and subclade (ANI 99.95%) levels is shown on branches and in the first lane, respectively. Subsequent lanes provide information on strain isolation, results of pathogenicity tests in cherry, the presence of genes encoding toxins, followed by the presences of genes encoding individual T3Es. Genes encoding AvrE1, HopAI1, HopW1, HopAG1, HopI1, HopBV1, HopB2, HopAA1, HopM.ShcM1 and HopAH1 were present in all strains although they are not shown. The presence of a T3E is marked in maroon if the gene encoding this T3E is found in a prophage sequence.

Using 99.95% ANI, the cut‐off commonly used for classification at strain level, isolates were classified into subclades. While most phylogenetic clades were divided into numerous distinct small subclades and variants, PG2d strains were grouped into a limited number of variants, with the majority of strains belonging to one of four common subclades (Figure 4, ring B). These results strongly suggest the clonal expansion of subclades of cherry pathogens in orchards. Although orchards in the Southeast were the main source of PG2d strains (Figure 3B), members of the common PG2d subclades were recovered from more than one region (Figure 5 and Table S3). The less abundant PG2d subclades (PG2d‐6, 9, 14 and 15; Table S3), which also contained domestic cherry pathogens, were only found in woodlands. Interestingly, subclade PG2d‐3 consisted of strains from both orchard and woodland.

Numerous subclades were found in PG2b. Of the 12 strains of PG2b confirmed as pathogens of cultivated cherry, five were from orchards and seven from woodland (Figure S3). Analysis of PG2b subclades within other clades presented in Figure 4 (ring B) showed that strains were typically recovered from more than one plant species (Table S4). Overall, there was no obvious indication of the adaptation of subclades of PG2b to a particular host plant.

### 2.3 Divergence of Effector and Toxin Repertoires in PG2b and PG2d

High frequencies of pathogenicity were found in members of PG2d in both cultivated cherry and wild cherry. Although PG2b is closely related to PG2d, most PG2b strains were not pathogenic, and such varied pathogenicity within PG2b and PG2d prompted a closer analysis of the repertoire of genes encoding T3Es and toxin biosynthesis in the two clades (Figure 5). Unlike PG2d, most strains within PG2b lacked the genes for syringolin A production. In strains of PG2d, pathogenicity to cultivated cherry was associated with the presence of effector genes *hopAW1*, *avrRpm1*, *hopAR1* and *hopBE1*, which were notably absent from PG2b strains. Only one strain of PG2d, which was of orchard origin, contained a T3E gene (*hopAR1*) from the common effector set within prophage sequences. This strain also carried *hopBK1* within a prophage.

### 2.4 Diversity and Origin of Subclades in PG2d

**FIGURE 6:**
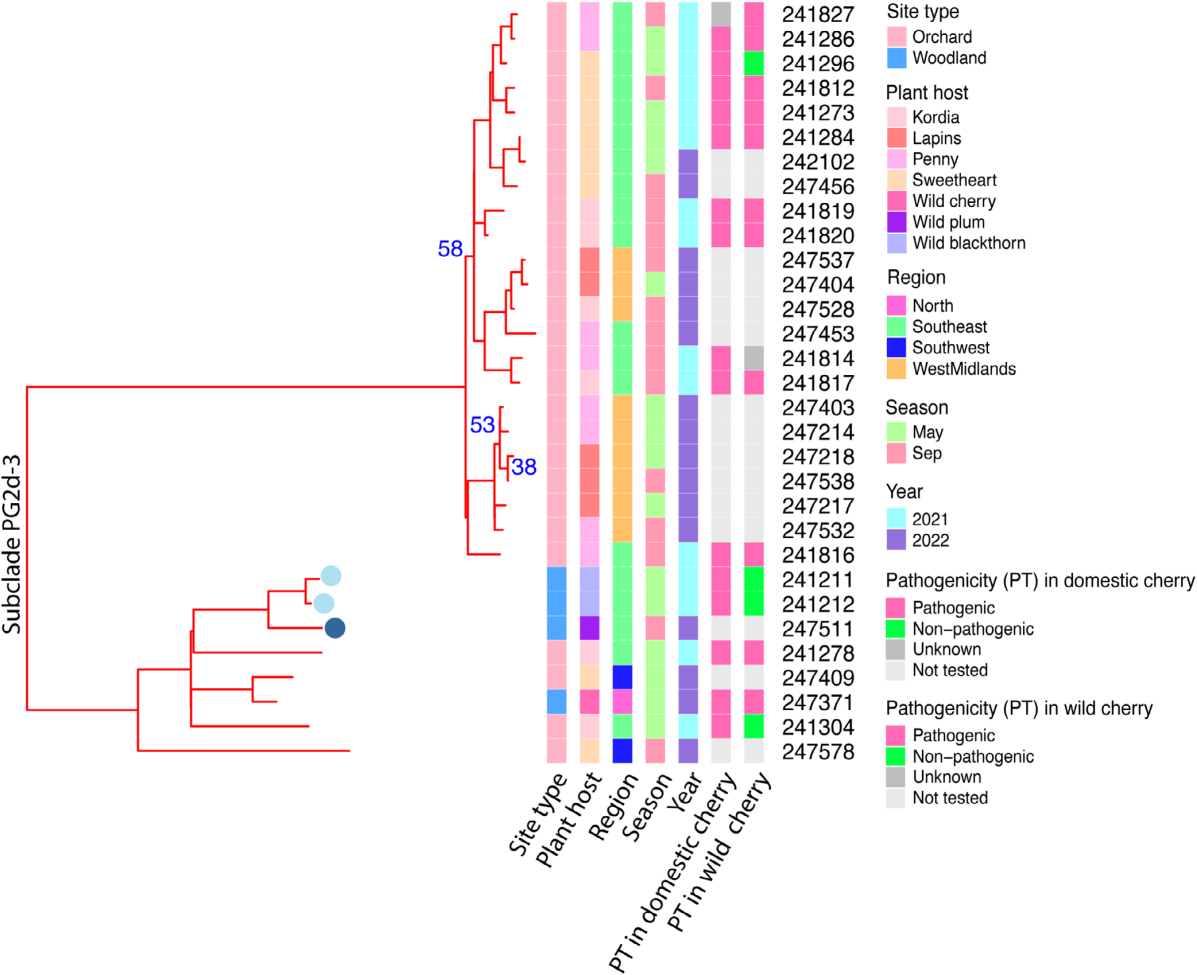
Maximum‐likelihood phylogeny of 31 *Pseudomonas syringae* strains from subclade PG2d‐3, constructed from 4897 core genes identified through pangenome analysis. The tree illustrates the relationship between strains isolated from orchard‐grown cherry trees and those recovered from wild plant hosts in adjacent woodlands. Bootstrap support values are shown for nodes with support below 70%. Strain isolation source (orchard or woodland, plant host, region, season and year) and pathogenicity test results on leaves of sweet cherry and wild cherry are shown. The six‐digit identifiers represent sequencing barcodes used in this study and can be used to match strain information across other Tables and Figures. A PG2d‐4 strain 241890 was used for re‐rooting the phylogenic tree (not shown). The three woodland strains that were found to contain specific prophages are circled, light blue for 49 kb prophage, dark blue for 63 kb prophage.

The near‐clonal subclade members of PG2d shared consistent patterns of T3E gene content but could be clearly separated by comparing the presence and absence of genes using pangenome analysis with Panaroo (Tonkin‐Hill et al. 2020) (Figure S6). To further investigate the diversity within these near‐clonal groups, we constructed a core genome phylogeny of all 31 strains assigned to subclade PG2d‐3, including those not tested for pathogenicity. The tree was built from 4897 core genes identified across 27 orchard‐derived strains and four isolates from woodland habitats. The resulting phylogeny suggested that the woodland strains had diverged from an orchard‐derived origin, with three of the woodland isolates (two recovered from blackthorn and one from wild plum) forming a distinct lineage (Figure 6). This pattern of divergence was also supported by a separate phylogeny based on core single‐nucleotide polymorphisms (SNPs) throughout genomes (Figure S7).

Further pangenome analysis of the 31 PG2d‐3 strains pinpointed 49 genes that are absent from orchard isolates but conserved in the three woodland strains that form a distinct lineage. Intriguingly, 38 of these genes were found in a predicted prophage region in each of the woodland strains (Table S5, highlighted in bold). The prophage sequences in the two blackthorn isolates were identical (49 kb), whereas the prophage in the wild plum isolate had a larger genome of 63 kb. The phages shared high identity across most core structural components but differed in their tail‐fibre genes. Genes within the prophage regions included potential contributors to bacterial adaptation including TetR family transcriptional regulators, serine/threonine phosphatase, and RecA‐like AAA+ ATPase (Table S6).

## 3 Discussion

### 3.1 Diversity of Epiphytic *P. syringae*

The presence of members of *P. syringae* phylogroups within diverse habitats has been reported previously (Monteil et al. 2016; Morris et al. 2013). Our results confirm the ubiquitous nature of *P. syringae* and illustrate how a similar range of PGs was recovered from cultivated cherry and all plant species sampled in nearby woodlands. However, there were exceptions as members of PG1b were not found in any orchards and the frequency of recovery of PGs from cultivated and wild plants was also very different. Comparisons using ANI 96% revealed the predominance of PG2d strains within orchards and PG2b and PG13a in nearby woodlands. We found a high frequency of PG13 strains in woodlands, particularly in the September samplings, but there was no clear evidence for adaptation to a particular woodland species. Members of PG13 have not been studied in detail although they are often reported in surveys of epiphytes (Morris et al. 2022). Our earlier ‘snapshot’ survey of orchards in England during May 2017 also found considerable phylogenetic diversity (Hulin et al. 2023). Most strains isolated from the Southwest and Southeast in 2017 were members of PG2d and PG10, respectively. The current study, during 2021 and 2022, extended sampling to Scotland and the northern site had strikingly fewer PG2d strains than found in other regions, notably the Southeast (Figure 3). Taken together, the survey results suggest that PG2d, which includes most strains pathogenic to cultivated and wild cherry, is prevalent in southern regions but rare in the North, suggesting a restricted or emerging distribution.

### 3.2 Factors Impacting Phyllosphere Colonisation

Populations of *P. syringae* are affected by various factors including the presence of other bacterial genera in the phyllosphere. Changes in epiphytic microbiomes over time and on different annual crops were reported by Copeland et al. (2015), who found that bacteria within the genera *Sphingomonas* and *Curtobacterium* dominated within the leaf microbiome. They also reported that the epiphytic microbiome was strongly influenced by genera commonly found in soil early in the season but over time more leaf‐specific taxa appeared such as *Pseudomonas* within the *Proteobacteria*. Soil‐borne species are unlikely to have influenced our analysis of tree leaves but they may have competed in the phyllosphere on wild strawberry which has a ground surface habit and had a distinctly different frequency of PGs compared with the trees. By contrast, strains from each of the tree species in the woodland had similar phylogenetic diversity. The impact of common microclimate on leaves within the tree canopy appears to have more impact on diversity than the plant species. Copeland et al. (2015) also found that microbiome phyla did not differ between plot location, crop species, or any of their respective cultivars. As mentioned above, we found differences between seasons, including a notable increase in strains from PG13 in September reflecting the change in dominant epiphytic microflora occurring throughout the year. Other studies on leaf surface microbiomes have demonstrated considerable host specificity. For example, in their examination of tree, shrub and herbaceous plants, Smets et al. (2023) found that host selection was an important force in microbiome assembly on lower but not on upper leaf surfaces. Microbial dispersal from surrounding vegetation has been reported to influence the phyllosphere microbiome assembly (Meyer et al. 2022; Meyer and Lindow 2025). Our focus on pseudomonads has not revealed clear host selection but highlights the important role of differing microenvironmental conditions on trees and ground‐restricted plants.

These observations raised an important question: which bacterial traits enable strains to persist on leaf surfaces independently of pathogenicity, a topic that remains poorly understood (Vorholt 2012; Wilson et al. 1999). Helmann et al. (2019, 2020) used random‐barcoded transposon sequencing in a PG2d strain (*P. syringae* B728a, which is not a cherry pathogen) to distinguish genes linked to epiphytic versus endophytic (pathogenic) fitness in bean. The presence of T3Es and also the production of the toxin syringomycin emerged as major virulence factors for bean, but not for multiplication on the leaf surface, which instead was associated with genes contributing to diverse metabolic pathways. These findings suggest that phyllosphere survival and virulence rely on distinct functional pathways.

### 3.3 Pathogenicity of *P. syringae* Strains Recovered From Leaf Surfaces

The high percentage of epiphytic isolates producing a clear HR on bean pods correlated with the presence of the canonical T‐PAI T3SS and effector genes, suggesting that the strong resistance reaction was an expression of effector‐triggered immunity (ETI). Given the ability of the strains to deliver T3Es, they may have the potential to invade plant hosts in which they are not recognised by ETI. The five bean pathogens from PG2b produced necrotic symptoms very similar to those recorded for the unrelated genomospecies *Pseudomonas viridiflava* within PGs 7 and 8 (Bartoli et al. 2014). Water‐soaking characteristic of symptoms produced by the bean halo blight bacterium *P. syringae* pv. *phaseolicola*, which is from PG3 (Arnold et al. 2011), was produced by four epiphytic strains, three within PG2d and a PG2b strain. In contrast to virulence to bean, strains pathogenic to cherry leaves were found in several other PGs, notably PG1a (Psm2), PG2a (*Pseudomonas cerasi*) and PG10. The detection of strains that were pathogenic on bean was unexpected, but reinforces the idea that outbreaks of plant diseases might have unexpected origins, with strains spreading from trees to herbaceous crops, like beans or vice versa.

The striking finding here was the dominance of pathogenic strains within PG2d on symptomless leaf surfaces in England. Many of these strains were classified as pathogenic to wild cherry (accession Howley Wood), despite this accession showing partial resistance to the PG1 strain Psm2 based on reduced necrosis in leaf infiltration assays (Hulin et al. 2022). The origin of pathogenic subclades of PG2d remains unknown. There is limited evidence to determine whether the initial invasion of orchards occurred from woodlands. Reservoirs of infection are usually discussed as sources spreading pathogens from the environment to plant crop or animal hosts. With PG2d we highlight the potential for disease to spread from commercial orchards and cause environmental impact in woodlands. In addition to their pathogenicity, PG2d strains are particularly well adapted to thrive as epiphytes under orchard conditions. Cherry canker symptoms were rarely observed in the orchards sampled.

### 3.4 Repertoires of Toxins and T3Es Related to Pathogenicity to Cherry

Compared with pathogenic PG2d, many strains from PG2b lacked genes for potential virulence factors—the toxin syringolin A and effectors HopAW1, AvrRpm1 and HopAR1. This group of effectors has previously been identified as part of the ‘*Prunus* cluster’, genes common in strains of *P. syringae* PG2d pathogenic to *Prunus* spp. (Vadillo‐Dieguez et al. 2024). Mutation of the cluster in PG2d strain Pss 9644 was found not to cause complete loss of pathogenicity but did cause reductions in virulence in cultivated cherry cv. Sweetheart and to a greater degree in ornamental cherry *Prunus incisa* (Vadillo‐Dieguez et al. 2024). The *hopAW1*, *avrRpm1* and *hopAR1* genes are not physically linked, indicating that they have not been inherited together by HGT involving plasmid or integrative and conjugative element exchange. Only *hopAR1* of the *Prunus* group of genes was located within a predicted prophage sequence and only in one of the PG2d strains sampled. In the study of Hulin, Armitage, et al. (2018), the *hopAR1* gene was also found in a prophage region of PG2 strains isolated from bean such as Pss syr2675, syr2676 and syr2682, and their sequences showed high similarity with the *hopAR1*‐containing prophage sequence from a Psm2 strain, suggesting prophage‐mediated horizontal gene transfer of *hopAR1* between members of Psm2 (PG1) and PG2. The presence of other effectors within prophage in our collection of *P. syringae* isolates (Figure S5) is consistent with bacteriophages contributing to HGT in the phyllosphere, although the extent of their involvement is unclear.

Apart from the presence of the ‘*Prunus* cluster’ and syringolin A production in pathogenic PG2d strains, there was no clear association between groups of effectors and pathogenicity to cherry. Variation in effector content was particularly marked amongst pathogenic strains within PG2b. Given that deletion of all effectors from Pss 9644 creates a nonpathogenic mutant (Vadillo‐Dieguez et al. 2024), the effector diversity observed probably reflects the occurrence of redundancy in effector functions (Kvitko et al. 2009). The redundancy of function of effector proteins in canker pathogens and indeed in many other plant pathogens will not be fully explained until the primary and combined functions of more effectors are determined. An intriguing aspect of bacterial virulence to plants is the finding that genes encoding effectors are retained despite their apparent individual lack of fitness benefit as assessed in the laboratory (Kvitko et al. 2009; Vadillo‐Dieguez et al. 2024).

### 3.5 Spread of Virulent PG2d in the UK

Although the PG2d strains examined here shared 99.95% ANI and were close to clonal, they were recovered from different locations within the UK (Figure 5). Similarly, diversification within PG2b has resulted in the generation of numerous subclades but there was no evidence for the clustering of strains into groups based on their host of origin. The prevalence of strains within PG2d in orchards has not yet been associated with an increase in the occurrence of bacterial canker. Historically, cherry canker has been linked to infection by Psm1 and Psm2 (Crosse 1966; Hulin et al. 2023; Ruinelli et al. 2019; Vicente et al. 2004), but it seems probable that future outbreaks will be caused by the build‐up of PG2d. All of the commercial orchards surveyed used polythene tunnels to cover cherry trees after flowering. The impact of such a common agronomic practice on the microbial ecology of the leaf surface and spread of canker should be examined. It is possible that in the absence of significant rain splash‐mediated dispersal, colonies of PG2d on the leaf surface are not able to spread to wounds and leaf scars that act as entry points for the bacterium into woody tissue.

Interestingly, within the genetically uniform strains of subclade PG2d‐3, our data reveal divergence between orchard and woodland populations, which could be associated with prophage‐mediated gene acquisition. The presence of potentially fitness‐enhancing factors in the prophage region, combined with their absence from orchard isolates, suggests that these elements may contribute to niche adaptation to the more complex woodland environment. Phage‐mediated adaptation has been increasingly recognised as a major driver of bacterial diversification and host range expansion (Sawada et al. 1999; Yamada et al. 2007; Hulin et al. 2023).

### 3.6 Importance and Limitations of Genomic Surveillance in Studies of *P. syringae*

Our experimental approach, as an example of genomic surveillance, has largely followed the proposals made by Weisberg et al. (2021). Our results address their conclusion that it is necessary to examine ecological relationships between nonpathogenic and pathogenic members of species. We have confirmed the value of high‐throughput WGS for surveys, though challenges related to data handling remain. For example, despite equal sampling efforts, fewer *P. syringae* strains were recovered from sites in Scotland. Nevertheless, clear differences were found between epiphytes in orchards and woodlands. Resolution of subclades within PG2d has provided insights into the emergence of highly virulent strains that are threats to both cherry orchards and environmental *Prunus* spp. We have demonstrated the potential value of sampling the epiphytic microbiome rather than focusing solely on infected tissue, an approach already adopted successfully by Hemara et al. (2025). A broader surveillance of the plant surface, coupled with a detailed understanding of pathogenicity factors, should lead to more accurate prediction of emerging strains of *P. syringae* likely to damage both agricultural crops and environmentally important plants. Our results already suggest that the identification of a strain as within PG2d with genes encoding the ‘*Prunus* cluster’ of effectors plus toxin synthesis (notably syringolin A) indicates a high probability of pathogenicity to *Prunus* spp. The identification of resistance to pathogenic PG2d strains should be a priority in the development of new cultivars of cherry.

## 4 Experimental Procedures

### 4.1 Bacteria and Media

The *P. syringae* strains used in this study are listed in Table S1 and were grown in King's medium B (KB; King et al. 1954) or lysogeny broth (LB) at 22°C. Cephalexin and cycloheximide were supplemented in the broth or agar media at the final concentration of 80 and 100 μg mL^−1^, respectively.

### 4.2 Experimental Design, Sampling and Bacterial Isolation

Seasonal samplings for *P. syringae* isolation were performed in both May and September in 2021 and 2022 in four regions in the UK: the Southeast (SE, Kent), Southwest (SW, Somerset and Dorset), West Midlands (WM, Staffordshire and Herefordshire) and North (N, Scotland). Within each region, orchards growing cherry ( *Prunus avium* ) varieties (Kordia, Lapins, Sweetheart and Penny) and two adjacent woodlands were sampled. In each woodland, six plant species increasingly unrelated to cherry were sampled, namely wild cherry ( *Prunus avium* ), wild plum (cherry plum [ *Prunus cerasifera* ] and bullace [*Prunus domestica*]), blackthorn ( *Prunus spinosa* ), hawthorn ( *Crataegus monogyna* ), wild strawberry (*Fragaria vesca*) and ash ( *Fraxinus excelsior* ). For each cherry cultivar or wild plant species, six trees were sampled, with two leaves collected from 2‐year‐old branches of each tree. The same branches were examined in repeated samplings. Details of the sampling strategy are shown in Tables 2 and S2.

**TABLE 2:** Numbers of trees of each cherry cultivar or wild plant sampled at each site in this study. Samples were collected from the same trees in all four samplings.

Orchards					
Region	Site	Sweetheart	Penny	Kordia	Lapins
SE	O1 (F.W Mansfield & Son)	6	6	6	0
	O2 (AC Hulme & Sons)	6	6	6	0
	O3 (Mount Ephraim Farm)	6	0	6	6
SW	O1 (Bagber)	6	6	0	6
	O2 (Whitehouse)	6	0	6	6
WM	O1 (Haygrove Ltd)	0	6	6	0
	O2 (Penrhos spirits)	0	6	6	6
	O3 (Lower Hope Fruit Ltd)	6	6	0	6
N	O1 (Castleton)	6	6	0	6
	O2 (Thomas Thomson)	6	0	6	6
	O3 (Marshall&Co.)	6	0	6	6

Woodlands							
Region	Site	Wild cherry	Blackthorn	Cherry plum/bullace	Hawthorn	Wild strawberry	Ash
SE	W1 (Hamlet)	6	6	5	6	6	6
	W2 (Saxten & Cage's)	6	6	6	6	6	6
SW	W1 (Gridler's Coppice)	6	6	6	6	6	6
	W2 (Black Rock)	6	6	5	6	6	4
WM	W1 (Beacon Hill)	6	6	0	6	6	6
	W2 (Thruxton Vallets)	6	6	6	6	1	6
N	W1 (Hally Burton)	6	6	6	6	6	6
	W2 (Westfield)	6	6	6	6	6	6

A whole leaf of approximately 10 cm in length was selected where possible. Multiple leaves of blackthorn and hawthorn were collected due to the smaller size of leaves. The leaf was rolled up and kept in a 15 mL Falcon tube. All the samples were stored in a prechilled cool box at 4°C before being processed within 24 h in most cases, and no later than 2 days after sampling. Epiphytes were isolated following the methods of Hulin et al. (2023) using phosphate‐buffered saline buffer to wash leaves. Putative *P. syringae* colonies were streaked on LB agar for purification post‐isolation.

### 4.3 High‐Throughput PCR and Agarose Gel Electrophoresis

Multiplex PCR with *P. syringae* species‐specific oligonucleotides (Psy_F: 5′‐ATGATCGGAGCGGACAAG‐3′ and Psy_R: 5′‐GCTCTTGAGGCAAGCACT‐3′ [Guilbaud et al. 2016]) and oligonucleotides targeting the *hrcC* gene of the canonical T‐PAI T3SS (HrcC_F1: 5′‐GCCTTTATTGTTGATCGGG‐3′ and HrcC_R2: 5′‐GTGTCGTTATASACRAACCACTGSAAGT‐3′; designed in this study based on the *hrcC* sequences of *P. syringae* members of PGs 1, 2, 3, 4 and 10) were used to identify *P. syringae* isolates and detect the presence of the canonical T‐PAI T3SS.

### 4.4 Whole Genome Sequencing

For each cultivar or plant species, four PCR‐identified candidate *P. syringae* isolates, each recovered from a different tree, were selected for WGS. Bacterial lysates were obtained by adding the lysis buffer provided by the sequencing facility to a diluted bacterial overnight culture (OD_600nm_ = 0.5) and stored at −80°C before being sent to MicrobesNG (Birmingham, UK) for DNA extraction, library preparation and whole genome sequencing. The strains sequenced in this study are shown in Table S1, and the sequences obtained in this study have been submitted to NCBI Sequence Read Archive under BioProject PRJNA1217355.

### 4.5 Genome Assembly and Annotation

Bioinformatics and computational analyses were performed on CropDiversity‐HPC (Percival‐Alwyn et al. 2024). Paired‐end raw reads were trimmed using Trim‐galore v. 0.6.10 (Kruger 2015) to remove adapter sequences and reads shorter than 75 bp. Trimmed reads were assessed using FastQC v. 0.12.1 (Andrews 2010) before genome assembly using SPAdes v. 4.0.0 (Bankevich et al. 2012). CheckM v. 1.2.3 (Parks et al. 2015) was used to filter for genomes with ≥ 95% completeness, ≤ 5% contamination and an N50 ≥ 40,000 bp. Gene annotations were performed using Bakta v. 1.11.0 (Schwengers et al. 2021). Functional annotation was obtained from BLASTx v. 2.16.0 (Altschup et al. 1990), PHASTEST v. 3.0 (for genes found in prophage regions) and HHpred (Zimmermann et al. 2018; web server build 57c8707149031cc9f8edceba362c71a3762bdbf8) if no information was obtained using the other tools.

### 4.6 *Pseudomonas syringae* Strain Identification

Published genomes of *P. syringae* species complex were downloaded from NCBI (taxonomic group ID 136849) in December 2022 and clustered into groups sharing 99.95% ANI using PYANI (Pritchard et al. 2016). One strain of each group was selected to produce a collection of *P. syringae* reference genomes. A newly sequenced strain was confirmed to be a member of the *P. syringae* species complex if the genome shared at least 95% ANI with a reference genome in the reference genome collection using FastANI v. 1.3.4 (Jain et al. 2018).

### 4.7 Phylogenetics, Pangenome Analyses and Prophage Identification

Pangenome analyses of *P. syringae* strains were performed using Panaroo v. 1.5.1 (Tonkin‐Hill et al. 2020) with genomes annotated using Bakta (Schwengers et al. 2021). Panaroo was run in strict cleaning mode with paralog merging enabled, and gene clusters were aligned using MAFFT (Multiple Alignment using Fast Fourier Transform). Gene presence‐absence matrices were generated using the following key parameters: a sequence identity threshold of 0.98, maximum length difference of 0.98, and a core genome threshold of 0.98. SNPs amongst PG2d strains were identified using Snippy v. 4.6.0 (Seemann 2015). Reads were mapped to the long read assembly of strain Pss 9097 (GCA_002905815.2), selected as the reference because it is the closest available genome to the PG2d isolates based on ANI. Using a more distant outgroup would have introduced excessive sequence divergence and reduced the size and reliability of the core SNP alignment; therefore, no outgroup was included. The resulting phylogeny was midpoint‐rooted, and bootstrap support values are shown at nodes with support below 70%. All the phylogeny trees were produced using IQTREE v. 2.2.2.7 (Minh et al. 2020) with 1000 bootstraps. For the phylogeny of PG2d‐3, a PG2d‐4 strain was used as an outgroup for re‐rooting. Because the outgroup produced a long branch, it was removed using the R package ggtree to improve visual clarity of the PG2d‐3 subclade.

### 4.8 Identification of Virulence‐Related Genes

The presence of genes encoding T3Es and toxins was identified using tBLASTn v. 2.16.0, following the approach described by Hulin, Armitage, et al. (2018). For T3E gene identification, the *P. syringae* effector sequence database published in Dillon, Almeida, et al. (2019) was used, applying a filtering threshold of ≥ 40% query length and ≥ 70% amino acid identity. Biosynthetic gene clusters encoding syringomycin, syringolin A, and syringopeptin were considered present if all cluster genes met thresholds of ≥ 50% query length coverage and ≥ 50% identity.

Genes of the canonical T‐PAI T3SS were also identified using tBLASTn v. 2.16.0 (Altschup et al. 1990), as per Hulin et al. (2020). The presence of the canonical T‐PAI T3SS was concluded for strains if at least 80% of the component genes listed for *P. syringae* pv. *tomato* DC3000 met thresholds of ≥ 50% query length coverage and ≥ 50% identity. Prophage sequences were detected using PhiSpy v. 3.7.8 (Akhter et al. 2012), and the predicted prophage regions were subsequently validated using PHASTEST v. 3.0 (Wishart et al. 2023), with T3E genes identified using the same criteria as described above.

### 4.9 Pathogenicity Tests

Putative *P. syringae* strains from two samplings in 2021 were selected for pathogenicity tests. A detached leaf infiltration assay was performed on cultivated cherry (Sweetheart) and partially susceptible wild cherry (Howley Wood) following the methods of Hulin, Mansfield, et al. (2018). Freshly picked leaves were infiltrated with bacterial suspension (2 × 10^6^ CFU mL^−1^). The cherry pathogen Pss 9644 and 10 mM MgCl_2_ were used as positive and negative controls, respectively.

Pathogenicity of environmental isolates was tested in French bean ( *P. vulgaris* ), distantly related to their plant of origin, as previously described Harper et al. (1987). Pods of field‐grown cultivars Canadian Wonder, Tendergreen, and Bush Blue Lake were inoculated by wounding with sterile sharply pointed toothpicks, the top of which carried bacterial colonies taken from LB agar plates. Inoculated pods were placed in clear plastic sandwich boxes base‐lined with moist tissue paper and incubated on the lab bench away from direct sunlight at 20°C–23°C. Symptoms were scored at 3–6 days post‐inoculation.

## Author Contributions

**Ziyue Zeng:** conceptualization, methodology, data curation, investigation, funding acquisition, writing – original draft, writing – review and editing, visualization, project administration, formal analysis. **John W. Mansfield:** conceptualization, investigation, supervision, funding acquisition, writing – review and editing, writing – original draft, methodology. **Andrea Vadillo‐Dieguez:** investigation, writing – review and editing, visualization, data curation, project administration, methodology. **John Connell:** data curation, investigation, formal analysis, methodology, writing – review and editing. **James Irvine:** writing – review and editing, methodology, data curation, investigation, project administration. **Michelle T. Hulin:** conceptualization, funding acquisition, writing – review and editing, supervision, methodology. **Fernando Duarte Frutos:** writing – review and editing, investigation, data curation. **Mojgan Rabiey:** investigation, writing – review and editing. **Nastasiya F. Grinberg:** formal analysis, writing – review and editing. **Richard J. Harrison:** conceptualization, funding acquisition, supervision, writing – review and editing, project administration. **Xiangming Xu:** writing – review and editing, conceptualization, supervision, project administration. **Robert W. Jackson:** writing – review and editing, conceptualization, supervision, investigation, funding acquisition.

## Conflicts of Interest

The authors declare no conflicts of interest.

## Supporting information


**Figure S1:** mpp70208‐sup‐0001‐FigureS1.docx.

**Figure S2:** mpp70208‐sup‐0002‐FigureS2.docx.

**Figure S3:** mpp70208‐sup‐0003‐FigureS3.docx.

**Figure S4:** mpp70208‐sup‐0004‐FigureS4.docx.

**Figure S5:** mpp70208‐sup‐0005‐FigureS5.docx.

**Figure S6:** mpp70208‐sup‐0006‐FigureS6.docx.

**Figure S7:** mpp70208‐sup‐0007‐FigureS7.docx.

**Table S1:** mpp70208‐sup‐0008‐TableS1.xlsx.

**Table S2:** mpp70208‐sup‐0009‐TableS2.docx.

**Table S3:** mpp70208‐sup‐0010‐TableS3.docx.

**Table S4:** mpp70208‐sup‐0011‐TableS4.docx.

**Table S5:** mpp70208‐sup‐0012‐TableS5.docx.

**Table S6:** mpp70208‐sup‐0013‐TableS6.docx.

## Data Availability

The datasets supporting the conclusions of this article are included within the article and the Supporting Information. The sequences obtained in this study have been submitted to NCBI Sequence Read Archive under BioProject PRJNA1217355. All assembled genomes (FASTA files) have been deposited on Figshare under the DOI: https://doi.org/10.6084/m9.figshare.30789062.
