# Supplementary material for: Genomic Surveillance of Epiphytic Pseudomonas syringae Highlights Shared Reservoirs and Cross‐Habitat Threats to Cherry Orchards and Nearby Woodland Plants
Source: Mol Plant Pathol. 2026 Feb 16;27(2):e70208. doi: 10.1111/mpp.70208 (PMC12910131; doi:10.1111/mpp.70208)
Supplement: Supplementary file 1 — Figure S1: mpp70208‐sup‐0001‐FigureS1.docx. [file MPP-27-e70208-s006.docx]

**
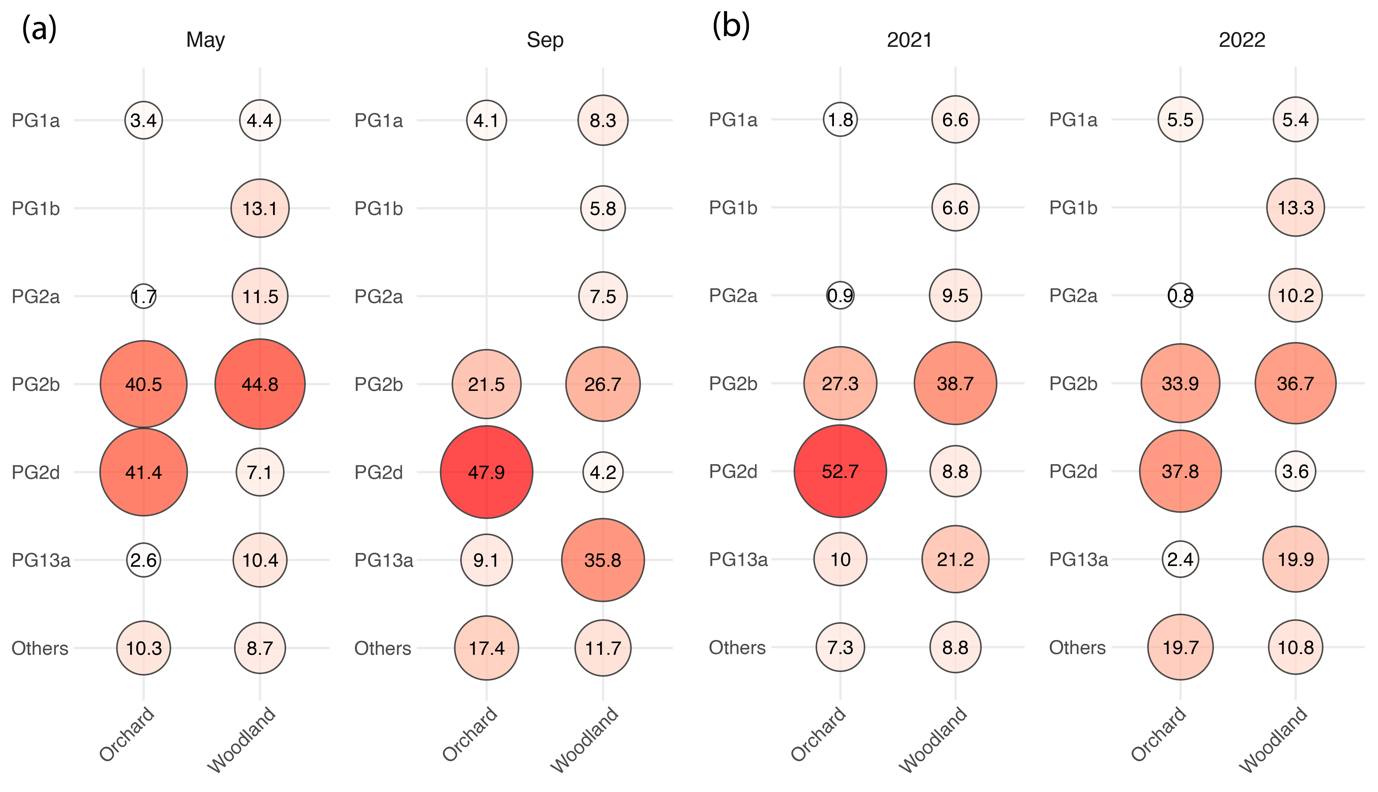
Figure S1** **Percentages of strains of each clade (ANI96%) isolated from orchards and woodlands from combined samplings in different (a) seasons and (b) years.** The numbers in the circles show the percentages that each clade takes up from the total numbers of strains isolated from orchards or woodlands in combined samplings. The size of the circle and the intensity of its red colour are proportional to the percentage inside the circle. Clades with small total numbers of strains were combined to be “Others”.
