# Supplementary material for: Genomic Surveillance of Epiphytic Pseudomonas syringae Highlights Shared Reservoirs and Cross‐Habitat Threats to Cherry Orchards and Nearby Woodland Plants
Source: Mol Plant Pathol. 2026 Feb 16;27(2):e70208. doi: 10.1111/mpp.70208 (PMC12910131; doi:10.1111/mpp.70208)
Supplement: Supplementary file 2 — Figure S2: mpp70208‐sup‐0002‐FigureS2.docx. [file MPP-27-e70208-s002.docx]

**
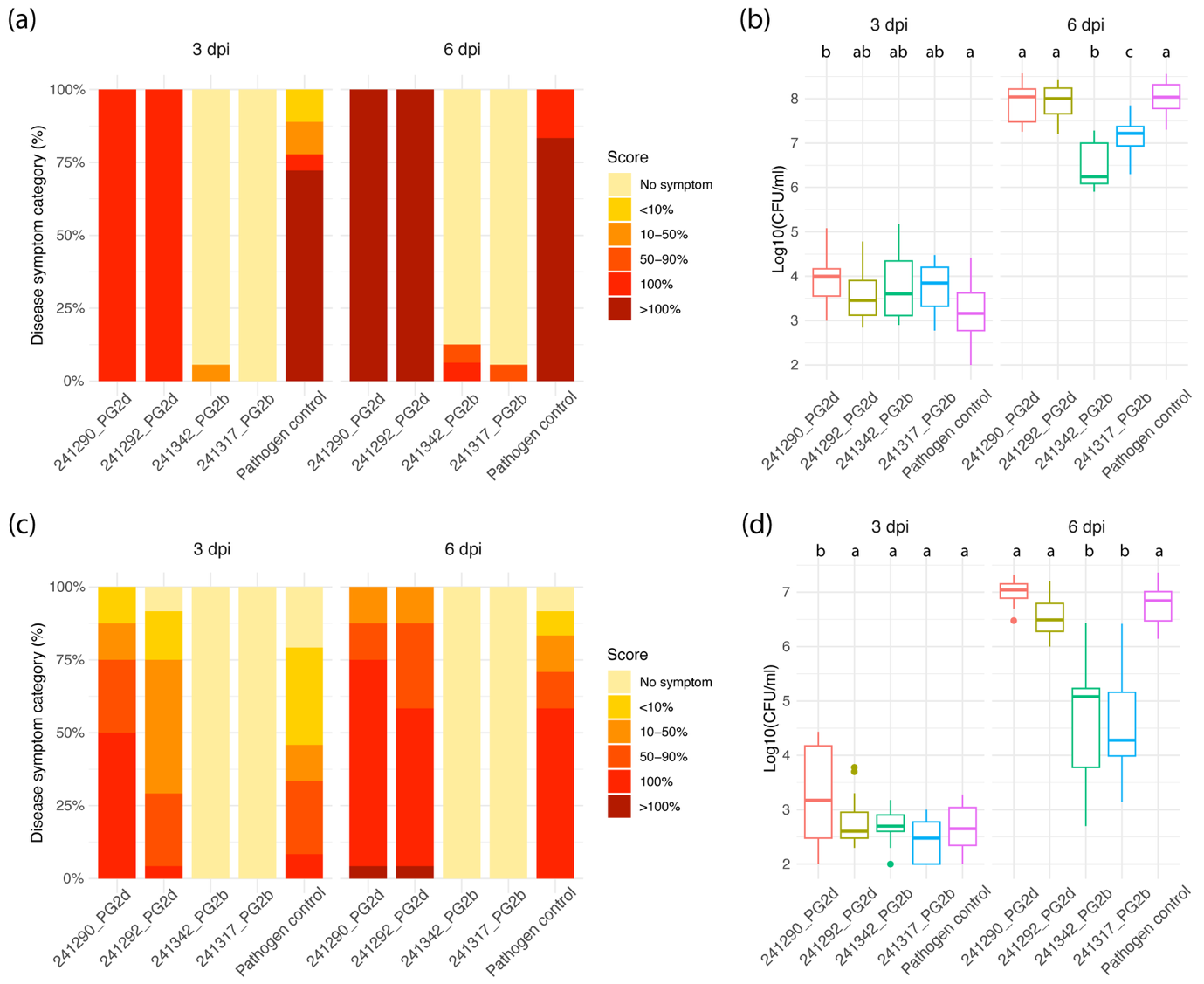
**

**Figure S2 Lesions in cherry leaves caused by proliferations of pathogens.** Lesion formation in detached leaves of (**a)** domestic cherry cv. Sweetheart and (**c)** a partially susceptible wild cherry accession (cv. Howley Wood) was assessed based on the percentage browning /blackening at the inoculation site: No symptom, <10%, 10%–50%, 50%–90%, 100% discolouration and symptoms spreading from the infiltrated area (>100%). Colony forming units (CFUs) of bacteria recovered from inoculation sites in (**b)** domestic cherry and (**d)** wild cherry. Data from at least three inoculation sites for each strain per timepoint were analysed. Box plots show minimum, first 25%, median, 75% and maximum counts with dots indicating outliers. Differences in log₁₀ CFU ml^-1^ among strains were tested by one-way ANOVA with Tukey HSD post-hoc comparisons (compact letter display). Bacterial overnight cultures (2$\times$10^6^ CFU ml^-1^) were used as inoculum. A known cherry pathogen *Pss* 9644 was used as pathogen control.
