# Supplementary material for: Genomic Surveillance of Epiphytic Pseudomonas syringae Highlights Shared Reservoirs and Cross‐Habitat Threats to Cherry Orchards and Nearby Woodland Plants
Source: Mol Plant Pathol. 2026 Feb 16;27(2):e70208. doi: 10.1111/mpp.70208 (PMC12910131; doi:10.1111/mpp.70208)
Supplement: Supplementary file 3 — Figure S3: mpp70208‐sup‐0003‐FigureS3.docx. [file MPP-27-e70208-s004.docx]

**
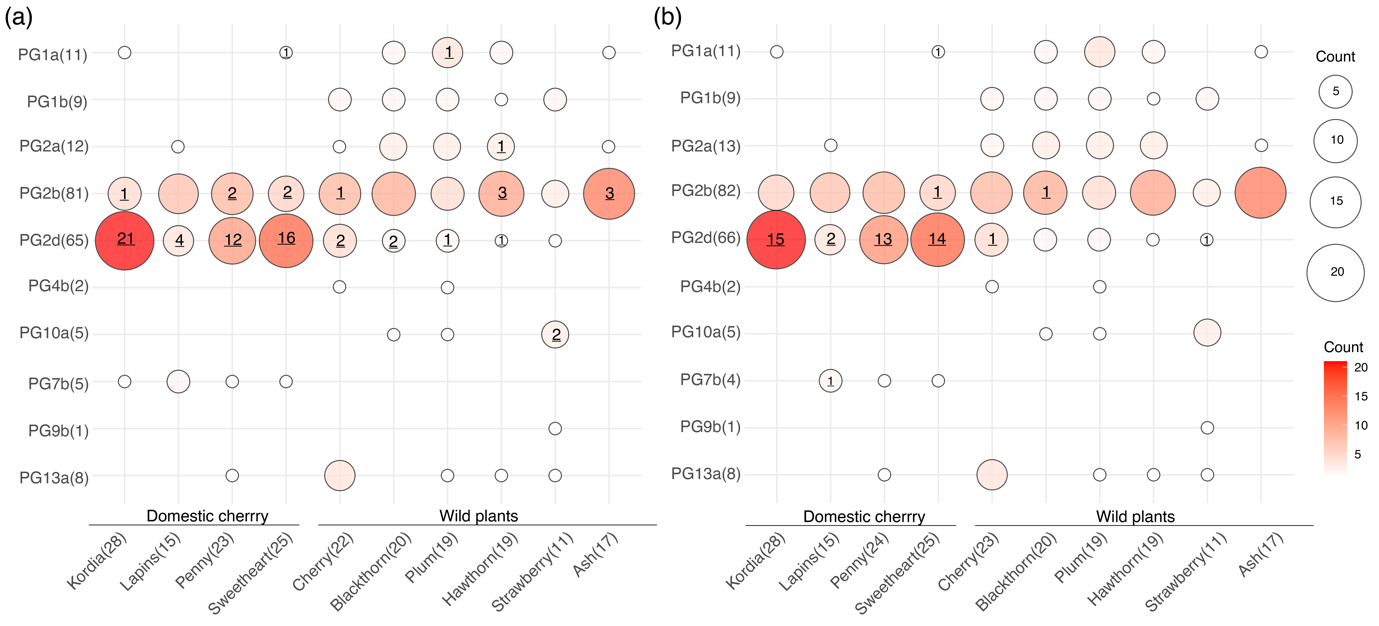
Figure S3 Numbers of cherry pathogens of each clade (ANI96%) isolated from different cultivars of domestic cherry or plant species in the woodlands in 2021.** Detached leaf assay in **(a)** Domestic cherry cv. Sweetheart and **(b)** Wild cherry (cv. Howley Wood). The size and intensity of the colour of circles visualise the total numbers of strains tested in each category. Total number of strains tested are shown in brackets, on the x-axis next to the plant name and the y-axis next to the clade name. The numbers in the circles show the number of pathogenic strains of each clade isolated from each cherry cultivar or wild plant species; no number is marked if no pathogen was found. Each bacterial strain was infiltrated on at least 3 different leaves of each type of cherry. Symptom development was scored at 3, 6 and 9 days after inoculation.
