# Supplementary material for: Genomic Surveillance of Epiphytic Pseudomonas syringae Highlights Shared Reservoirs and Cross‐Habitat Threats to Cherry Orchards and Nearby Woodland Plants
Source: Mol Plant Pathol. 2026 Feb 16;27(2):e70208. doi: 10.1111/mpp.70208 (PMC12910131; doi:10.1111/mpp.70208)
Supplement: Supplementary file 4 — Figure S4: mpp70208‐sup‐0004‐FigureS4.docx. [file MPP-27-e70208-s013.docx]

**
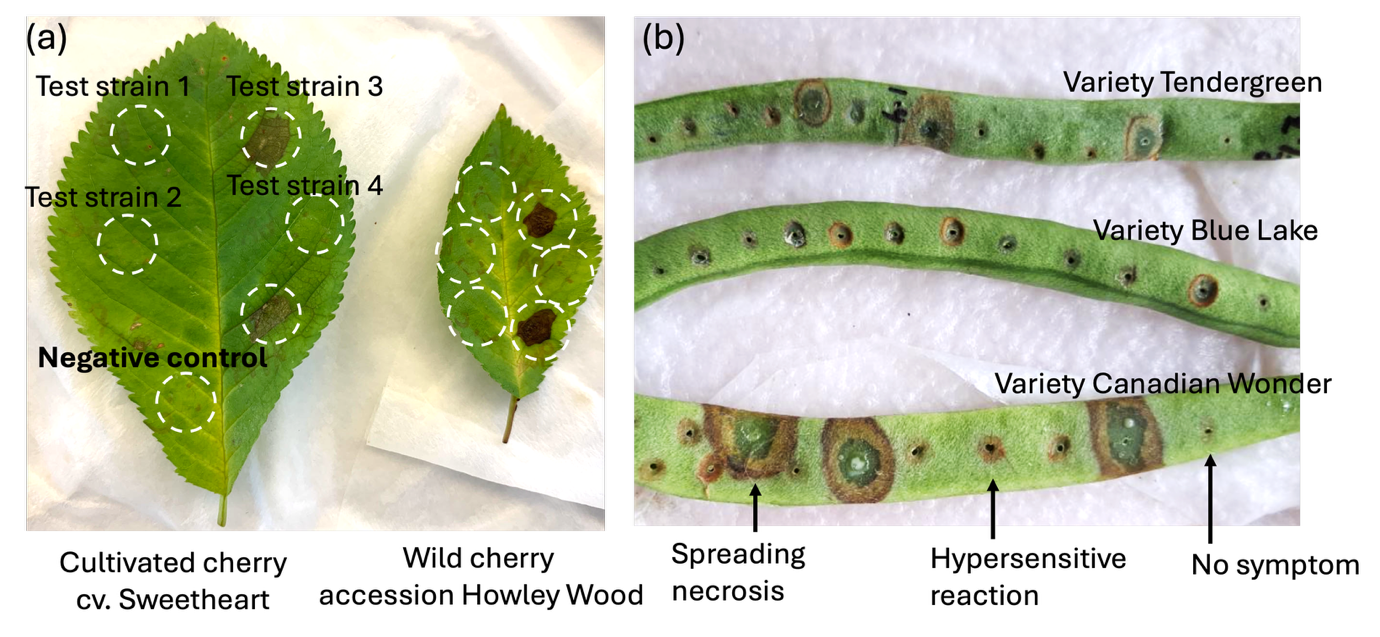
Figure S4 Disease symptoms in cherry and bean. (a)** Detached cherry leaf infiltration assay. **(b)** Bean pod stab inoculation. For leaf infiltration assay, bacterial culture (2$\times$10^6^ CFU ml^-1^) was used. Disease symptoms were scored at 3, 6 and 9 days post inoculation. Fresh bacterial colonies were picked by sterile toothpick for stab inoculation in beans. Disease symptoms were observed at 3-6 days post inoculation.
