# Supplementary material for: Genomic Surveillance of Epiphytic Pseudomonas syringae Highlights Shared Reservoirs and Cross‐Habitat Threats to Cherry Orchards and Nearby Woodland Plants
Source: Mol Plant Pathol. 2026 Feb 16;27(2):e70208. doi: 10.1111/mpp.70208 (PMC12910131; doi:10.1111/mpp.70208)
Supplement: Supplementary file 5 — Figure S5: mpp70208‐sup‐0005‐FigureS5.docx. [file MPP-27-e70208-s007.docx]

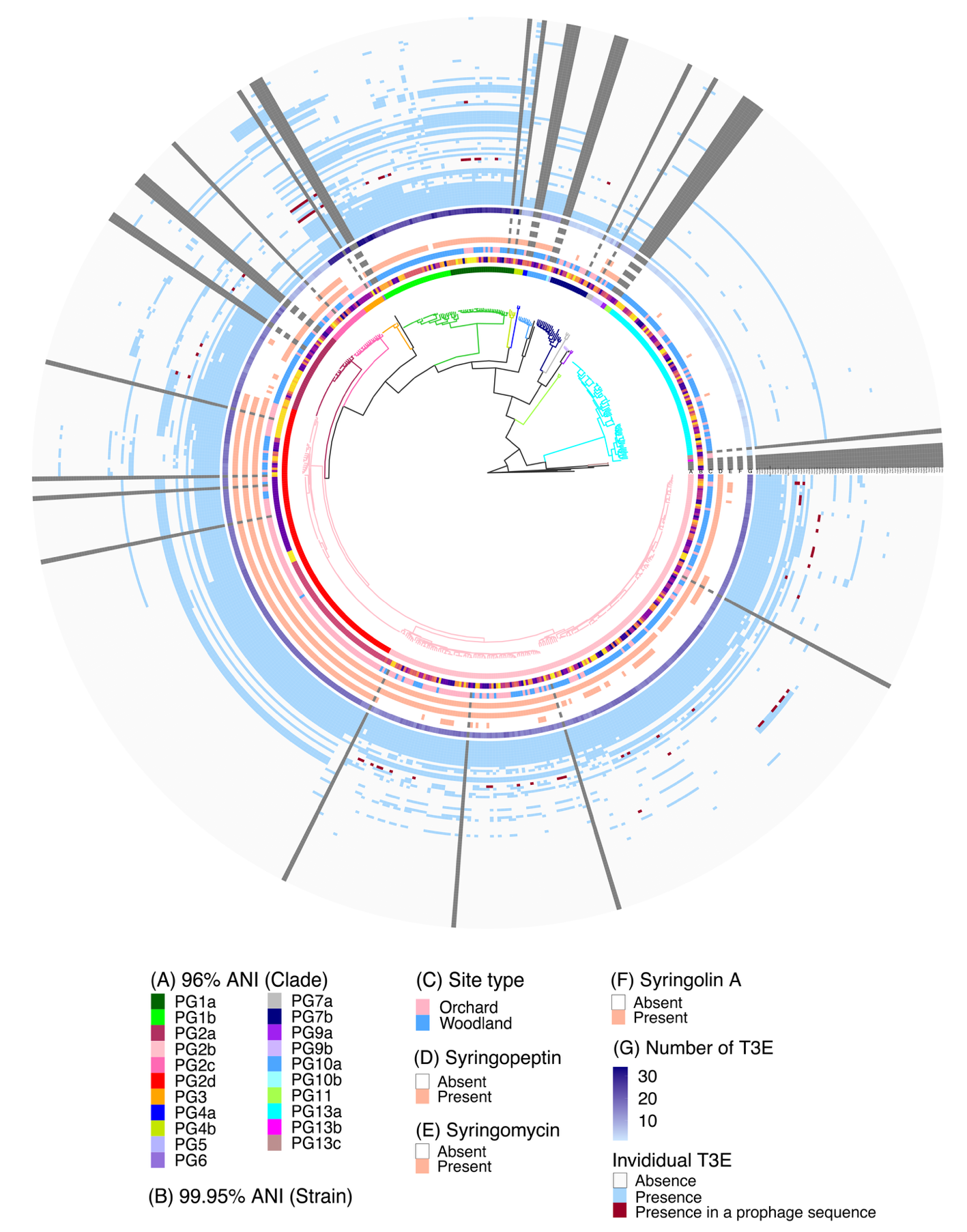
**Figure S5 Presence of genes encoding Type III effector proteins and toxin biosynthesis in all *P. syringae* strains recovered in this study.** A maximum likelihood core genome phylogeny tree of 540 *Ps* strains isolated from samplings and 39 reference strains. Lanes A and B show classification at the clade (ANI 96%) and subclade (ANI 99.95%) levels, respectively. Lane C shows site type of strain origin. Lanes D-H show the presence of genes encoding toxins, the presence of genes encoding the canonical T-PAI T3SS and the number of genes encoding T3Es, followed by the presences of genes encoding individual T3Es.
