# Supplementary material for: Genomic Surveillance of Epiphytic Pseudomonas syringae Highlights Shared Reservoirs and Cross‐Habitat Threats to Cherry Orchards and Nearby Woodland Plants
Source: Mol Plant Pathol. 2026 Feb 16;27(2):e70208. doi: 10.1111/mpp.70208 (PMC12910131; doi:10.1111/mpp.70208)
Supplement: Supplementary file 6 — Figure S6: mpp70208‐sup‐0006‐FigureS6.docx. [file MPP-27-e70208-s010.docx]

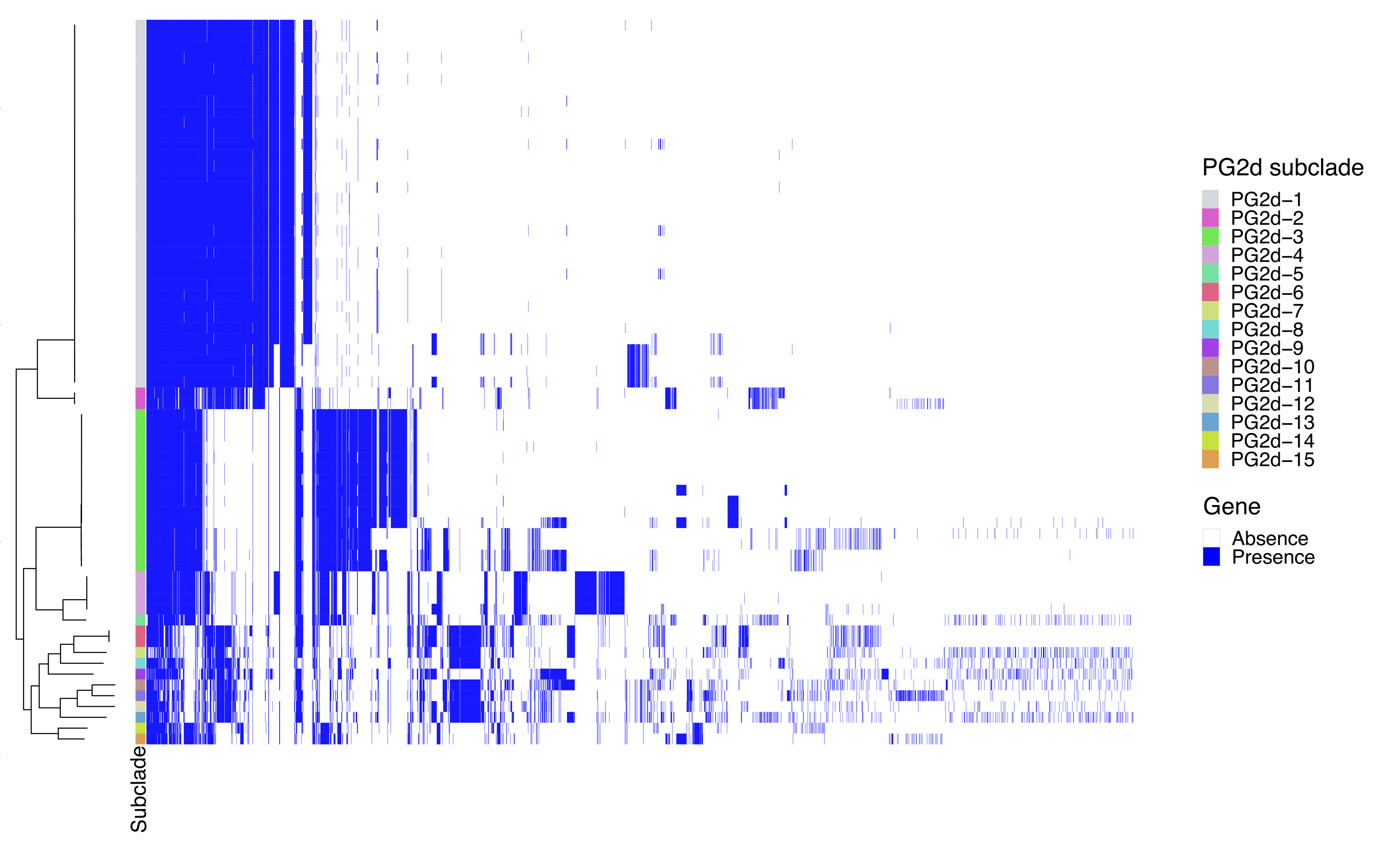


**Figure S6 Pangenome analysis using Panaroo to identify genes distinguishing subclades within phylogroup 2d strains.** The phylogenetic dendrogram is a subtree extracted from the core genome phylogeny in **Figure 1**, including PG2d strains that were isolated from 2021 and tested for virulence, as shown in **Figure 5**. The gene presence and absence matrix illustrates the diversity of PG2d. Subclades are numbered as in **Table S3.**
