# Supplementary material for: Genomic Surveillance of Epiphytic Pseudomonas syringae Highlights Shared Reservoirs and Cross‐Habitat Threats to Cherry Orchards and Nearby Woodland Plants
Source: Mol Plant Pathol. 2026 Feb 16;27(2):e70208. doi: 10.1111/mpp.70208 (PMC12910131; doi:10.1111/mpp.70208)
Supplement: Supplementary file 7 — Figure S7: mpp70208‐sup‐0007‐FigureS7.docx. [file MPP-27-e70208-s005.docx]

**
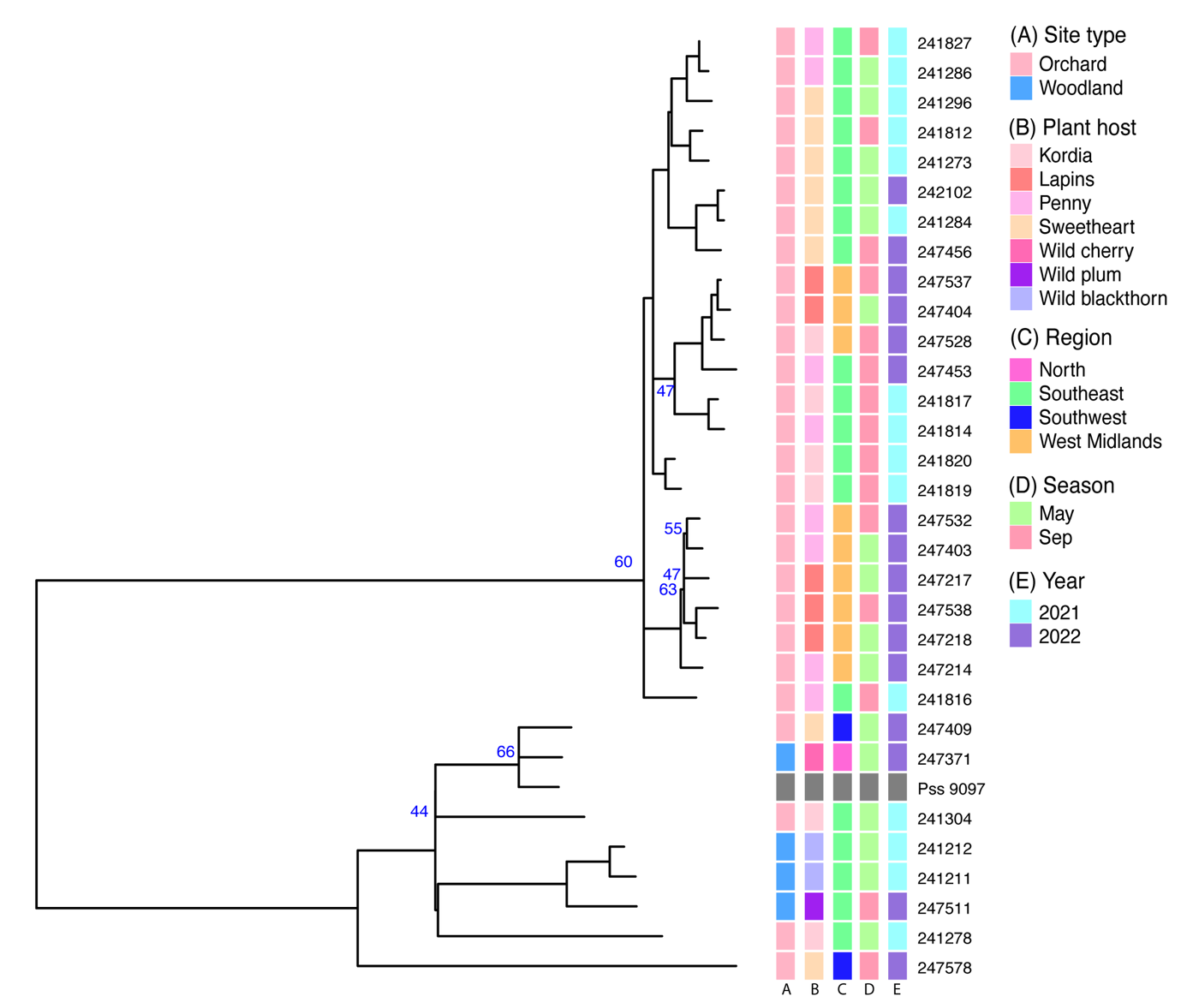
**

**Figure S7 Maximum-likelihood phylogeny of 31 *P. syringae* strains from subclade PG2d-3 based on core single nucleotide polymorphisms.** The tree shows the genetic relationships between strains isolated from orchard-grown cherry trees and those recovered from wild plant hosts in adjacent woodlands. The core single-nucleotide-polymorphism phylogeny was constructed using Snippy, with the long read *Pss* 9097 (GCA_002905815.2) sequence serving as a reference genome. The tree was midpoint-rooted. Bootstrap support values are indicated at nodes with support below 70%. Metadata for each strain, including isolation source (orchard or woodland), plant host, geographic region, sampling season and year as well as results of pathogenicity tests on sweet cherry and wild cherry leaves are annotated alongside the tips. Within the woodland lineage, blackthorn isolates are 241211 and 241212, and the wild plum isolate is 247511. The six-digit identifiers represent sequencing barcodes used in this study and can be used to match strain information across other tables and figures.
