# Supplementary material for: Genomic Surveillance of Epiphytic Pseudomonas syringae Highlights Shared Reservoirs and Cross‐Habitat Threats to Cherry Orchards and Nearby Woodland Plants
Source: Mol Plant Pathol. 2026 Feb 16;27(2):e70208. doi: 10.1111/mpp.70208 (PMC12910131; doi:10.1111/mpp.70208)
Supplement: Supplementary file 9 — Table S2: mpp70208‐sup‐0009‐TableS2.docx. [file MPP-27-e70208-s012.docx]

**Table S2 Numbers of strains isolated/sequenced/confirmed to be *P. syringae* based on genomic sequences.** Four strains were isolated from the two leaves collected from the same tree. To avoid sequencing clonal strains from the same tree, only one PCR-confirmed *Ps* strain from each tree was selected for whole genome sequencing. SE - Southeast, SW - Southwest, WM - West Midlands, N - North.

| **Region** | **Domestic cherry cultivars** | | | | **Wild plant species** | | | | | | **Total** |
| --- | --- | --- | --- | --- | --- | --- | --- | --- | --- | --- | --- |
|  | **Sweetheart** | **Penny** | **Kordia** | **Lapins** | **Cherry** | **Blackthorn** | **Plum** | **Hawthorn** | **Strawberry** | **Ash** |  |
| **SE** | 288/43/34 | 192/32/23 | 288/45/34 | 96/15/8 | 192/32/14 | 192/24/9 | 176/30/16 | 192/27/15 | 192/26/5 | 192/28/14 | 2000/302/172 |
| **SW** | 192/32/27 | 96/15/13 | 96/16/11 | 192/32/22 | 192/29/18 | 192/29/15 | 176/29/14 | 192/30/17 | 192/25/10 | 160/21/9 | 1680/258/156 |
| **WM** | 96/12/1 | 288/33/10 | 192/30/15 | 192/20/10 | 192/28/16 | 192/29/16 | 96/16/11 | 192/30/18 | 112/16/7 | 192/30/18 | 1744/244/122 |
| **N** | 288/46/10 | 96/16/3 | 192/27/8 | 288/39/8 | 192/30/8 | 192/27/11 | 192/30/9 | 192/28/12 | 192/25/6 | 192/29/15 | 2016/297/90 |
| **Total** | 864/133/72 | 672/96/49 | 768/118/68 | 768/106/48 | 768/119/56 | 768/109/51 | 640/105/50 | 768/115/62 | 688/92/28 | 736/108/56 | 7440/1101/540 |
