# Supplementary material for: Genomic Surveillance of Epiphytic Pseudomonas syringae Highlights Shared Reservoirs and Cross‐Habitat Threats to Cherry Orchards and Nearby Woodland Plants
Source: Mol Plant Pathol. 2026 Feb 16;27(2):e70208. doi: 10.1111/mpp.70208 (PMC12910131; doi:10.1111/mpp.70208)
Supplement: Supplementary file 10 — Table S3: mpp70208‐sup‐0010‐TableS3.docx. [file MPP-27-e70208-s003.docx]

**Table S3 Features of subclades within PG2d sharing 99.95% average nucleotide identities.** Only strains selected for pathogenicity assay are included. Information was summarised from **Figure 5**, with full metadata available in **Table S1**.

| **Subclade** | **Number of strains** | **Orchard (O) or Woodland (W) origin^a^** | **Region of origin** | **Pathogenicity^b^ to domestic cherry** | **Pathogenicity^b^ to wild cherry** | **Number of strains containing T3E genes *hopAW1*, *hopAR1 & AvrRpm1*** | **Number of strains containing T3E gene *hopBE1*** |
| --- | --- | --- | --- | --- | --- | --- | --- |
| PG2d-1 | 34 | O = 34 | N = 1,  SE = 25, SW = 8 | P = 33, Unkn= 1 | P = 29, nP = 4,  Int = 1 | 34 | 0 |
| PG2d-2 | 2 | O = 2 | SE = 1, SW = 1 | P = 2 | P = 1, nP = 1 | 2 | 0 |
| PG2d-3 | 15 | O = 13, W = 2 ^(2 blackthorn)^ | SE = 15 | P = 14, Unkn = 1 | P = 10, nP = 4,  Unkn = 1 | 15 | 15 |
| PG2d-4 | 4 | O = 4 | SE = 1,  SW = 2, WM = 1 | P = 4 | P = 2, nP = 1,  Int = 1 | 4 | 4 |
| PG2d-5 | 1 | O = 1 | SW = 1 | P = 1 | P = 1 | 1 | 1 |
| PG2d-6 | 2 | W = 2 ^(1 cherry, 1 hawthorn)^ | WM = 2 | P = 1, nP = 1 | nP = 2 | 0 | 2 |
| PG2d-7 | 1 | W = 1 ^(plum)^ | SE = 1 | nP = 1 | nP = 1 | 0 | 0 |
| PG2d-8 | 1 | O = 1 | SE = 1 | P = 1 | nP = 1 | 0 | 1 |
| PG2d-9 | 1 | W = 1 ^(cherry)^ | SE = 1 | P = 1 | P = 1 | 0 | 0 |
| PG2d-10 | 1 | W = 1 ^(cherry)^ | SE = 1 | nP = 1 | nP = 1 | 0 | 0 |
| PG2d-11 | 1 | O = 1 | SE = 1 | Int = 1 | P = 1 | 0 | 0 |
| PG2d-12 | 1 | W = 1 ^(strawberry)^ | SE = 1 | Int = 1 | P = 1 | 0 | 1 |
| PG2d-13 | 1 | W = 1 ^(cherry)^ | SE = 1 | Int = 1 | nP = 1 | 0 | 1 |
| PG2d-14 | 1 | W = 1 ^(cherry)^ | SE = 1 | P = 1 | nP = 1 | 1 | 1 |
| PG2d-15 | 1 | W = 1 ^(plum)^ | SE = 1 | P = 1 | nP = 1 | 1 | 1 |

^a^ Wild plant host is marked in brackets.

^b^ P for pathogenic, nP for non-pathogenic, Int for intermediate, Unkn for unkown.
