# Supplementary material for: Genomic Surveillance of Epiphytic Pseudomonas syringae Highlights Shared Reservoirs and Cross‐Habitat Threats to Cherry Orchards and Nearby Woodland Plants
Source: Mol Plant Pathol. 2026 Feb 16;27(2):e70208. doi: 10.1111/mpp.70208 (PMC12910131; doi:10.1111/mpp.70208)
Supplement: Supplementary file 11 — Table S4: mpp70208‐sup‐0011‐TableS4.docx. [file MPP-27-e70208-s008.docx]

**Table S4 Features of subclades (ANI 99.95%) of all clades that include strains isolated from both orchards and woodlands, including strains that were not selected for pathogenicity assay.** Subclade number was assigned based on the classification of all *P. syringae* strains, not within the clade. Full metadata for all strains are provided in **Table S1**.

| **Clade** | **Subclade No.** | **Site type** | **Region** | **Plant host** | **Number of strains** |
| --- | --- | --- | --- | --- | --- |
| PG1a | No.90 | O | WM | Domestic cherry | 2 |
|  |  | W | SW | Wild plum | 1 |
| PG2b | No.24 | O | N | Domestic cherry | 1 |
|  |  | W | SE | Wild blackthorn | 1 |
|  | No.29 | O | SE | Domestic cherry | 1 |
|  |  | W | SE | Wild ash | 1 |
|  | No.124 | O | SW | Domestic cherry | 1 |
|  |  |  | WM | Domestic cherry | 1 |
|  |  | W | SW | Wild cherry | 1 |
|  | No.169 | O | WM | Domestic cherry | 1 |
|  |  | W | SW | Wild cherry | 1 |
|  | No.177 | O | N | Domestic cherry | 1 |
|  |  | W | SW | Wild hawthorn | 1 |
|  |  |  | WM | Wild hawthorn | 1 |
| PG2d | No.3 (PG2d-1) | O | N | Domestic cherry | 1 |
|  |  |  | SE | Domestic cherry | 33 |
|  |  |  | SW | Domestic cherry | 19 |
|  |  |  | WM | Domestic cherry | 5 |
|  |  | W | SE | Wild ash | 1 |
|  | No.17 (PG2d-3) | O | SE | Domestic cherry | 16 |
|  |  |  | SW | Domestic cherry | 2 |
|  |  |  | WM | Domestic cherry | 9 |
|  |  | W | N | Wild cherry | 1 |
|  |  |  | SE | Wild blackthorn | 2 |
|  |  |  | SE | Wild plum | 1 |
| PG13a | No.11 | O | SW | Domestic cherry | 1 |
|  |  | W | SW | Wild cherry | 1 |
