# Supplementary material for: Genomic Surveillance of Epiphytic Pseudomonas syringae Highlights Shared Reservoirs and Cross‐Habitat Threats to Cherry Orchards and Nearby Woodland Plants
Source: Mol Plant Pathol. 2026 Feb 16;27(2):e70208. doi: 10.1111/mpp.70208 (PMC12910131; doi:10.1111/mpp.70208)
Supplement: Supplementary file 12 — Table S5: mpp70208‐sup‐0012‐TableS5.docx. [file MPP-27-e70208-s001.docx]

**Table S5 Bacteriophages predicted by PHASTEST in strains 241211 (blackthorn), 241212 (blackthorn) and 247511 (wild plum).** The table includes information on prophage location, contig coordinates, completeness, GC content, and the closest matching reference phage. Phage regions marked in bold and asterisk contain the 38 woodland lineage-specific genes identified in this study.

| **Region** | **Start** | **End** | **Size (kb)** | **Contig** | **Contig Start** | **Contig End** | **GC (%)** | **Completeness** | **Most Common Phage** |
| --- | --- | --- | --- | --- | --- | --- | --- | --- | --- |
| ***241211_1** | **105381** | **154779** | **49** | **contig_1** | **1** | **724807** | **58.17** | **intact** | **PHAGE_Pseudo_phiPSA1_NC_024365** |
| 241211_2 | 1197466 | 1229984 | 33 | contig_3 | 1130091 | 1491279 | 55.05 | incomplete | PHAGE_Xylell_Xfas53_NC_013599 |
| 241211_3 | 1229277 | 1284874 | 56 | contig_3 | 1130091 | 1491279 | 58.23 | intact | PHAGE_Pseudo_YMC11/02/R656_NC_028657 |
| 241211_4 | 2795330 | 2819358 | 24 | contig_8 | 2768279 | 3035707 | 57.96 | intact | PHAGE_Vibrio_vB_VpaM_MAR_NC_019722 |
| 241211_5 | 4999993 | 5018918 | 19 | contig_22 | 4944798 | 5033461 | 59.99 | intact | PHAGE_Salmon_118970_sal3_NC_031940 |
| ***241212_1** | **123141** | **172539** | **49** | **contig_1** | **1** | **1134060** | **58.17** | **intact** | **PHAGE_Pseudo_phiPSA1_NC_024365** |
| 241212_2 | 955721 | 1006107 | 50 | contig_1 | 1 | 1134060 | 57.59 | intact | PHAGE_Pseudo_YMC11/02/R656_NC_028657 |
| 241212_3 | 994489 | 1028923 | 34 | contig_1 | 1 | 1134060 | 56.08 | incomplete | PHAGE_Pseudo_phiPSA1_NC_024365 |
| 241212_4 | 2678966 | 2702994 | 24 | contig_6 | 2651915 | 2919343 | 57.96 | intact | PHAGE_Vibrio_VP58.5_NC_027981 |
| 241212_5 | 4805644 | 4824569 | 19 | contig_18 | 4738430 | 4839112 | 59.99 | intact | PHAGE_Salmon_118970_sal3_NC_031940 |
| ***247511_1** | **746054** | **808822** | **63** | **contig_1** | **1** | **932001** | **57.69** | **intact** | **PHAGE_Pseudo_phiPSA1_NC_024365** |
| 247511_2 | 959279 | 983307 | 24 | contig_2 | 932002 | 1342874 | 57.96 | intact | PHAGE_Vibrio_VP58.5_NC_027981 |
| 247511_3 | 3666482 | 3698823 | 32 | contig_11 | 3569800 | 3771201 | 55.21 | incomplete | PHAGE_Pseudo_phiPSA1_NC_024365 |
| 247511_4 | 3698116 | 3753713 | 56 | contig_11 | 3569800 | 3771201 | 58.23 | intact | PHAGE_Pseudo_YMC11/02/R656_NC_028657 |
| 247511_5 | 5134760 | 5153685 | 19 | contig_22 | 5120264 | 5208880 | 59.99 | intact | PHAGE_Salmon_118970_sal3_NC_031940 |
