# Supplementary material for: Genomic Surveillance of Epiphytic Pseudomonas syringae Highlights Shared Reservoirs and Cross‐Habitat Threats to Cherry Orchards and Nearby Woodland Plants
Source: Mol Plant Pathol. 2026 Feb 16;27(2):e70208. doi: 10.1111/mpp.70208 (PMC12910131; doi:10.1111/mpp.70208)
Supplement: Supplementary file 13 — Table S6: mpp70208‐sup‐0013‐TableS6.docx. [file MPP-27-e70208-s009.docx]

**Table S6** **Predicted functions and lengths of 49 woodland lineage-specific genes.** Gene groups were assigned based on the pangenome analysis of 31 *P. syringae* strains from subclade PG2d-3. Aside from the first 11 genes, the remaining 38 genes were found in a prophage region predicted using PHASTEST. Functional annotations were obtained using Bakta, BLAST and PHASTEST (for genes found in a prophage region) and HHpred if no information was obtained using those three tools.

| **Group** | **Length (bp)** | **Phage/bacterial gene predicted by PHASTEST** | **Function** | **Annotation tool** |
| --- | --- | --- | --- | --- |
| group_2208 | 158 | Not in a predicted prophage region | Hypothetical protein ALO72_200078 [*Pseudomonas syringae* pv. *delphinii*] | Blastx |
| group_2162 | 236 | Not in a predicted prophage region | Deoxyhypusine hydroxylase [Homo sapiens] | HHpred |
| group_2175 | 215 | Not in a predicted prophage region | Protein spackle [Escherichia virus T4] | HHpred |
| group_2190 | 194 | Not in a predicted prophage region | Hypothetical protein [Multispecies: *Pseudomonas syringae* group] | Blastx |
| group_2248 | 581 | Not in a predicted prophage region | DSBA oxidoreductase | Bakta |
| group_1984 | 449 | Not in a predicted prophage region | Hypothetical protein [Multispecies: *Pseudomonas*] | Blastx |
| group_2133 | 743 | Not in a predicted prophage region | Putative transporter [*Pseudomonas syringae* pv. *tomato* DC3000] | Blastx |
| group_2168 | 224 | Not in a predicted prophage region | DUF6555 family protein [Multispecies: *Pseudomonas*] | Blastx |
| group_2033 | 1619 | Not in a predicted prophage region | Methyl-accepting chemotaxis protein [*Pseudomonas viridiflava*] | Blastx |
| group_2264 | 560 | Not in a predicted prophage region | GNAT family N-acetyltransferase [Multispecies: *Pseudomonas*] | Blastx |
| group_2222 | 89 | Not in a predicted prophage region | Hypothetical protein [*Pseudomonas* sp. PvP009] | Blastx |
| group_582 | 734 | Phage | Metallophosphoesterase [*Pseudomonas syringae*] | Blastx |
| group_792 | 413 | Phage | HNH endonuclease [*Pseudomonas alliivorans*] | Blastx |
| group_865 | 332 | Bacterial | Hypothetical protein AO278_08170 [*Pseudomonas syringae* pv. *syringae*] | Blastx |
| group_541 | 788 | Phage | Conserved membrane protein [*Sulfolobus acidocaldarius* DSM 639] | HHpred |
| group_147 | 1469 | Phage | DNA cytosine methyltransferase [*Pseudomonas syringae*] | Blastx |
| group_668 | 596 | Phage | Transcription initiation factor IIE, alpha subunit [Homo sapiens] | HHpred |
| group_837 | 359 | Bacterial | Transcriptional regulator FleQ [*Pseudomonas aeruginosa*] | HHpred |
| group_492 | 866 | Phage | ATP-binding protein [*Pseudomonas syringae*] | Blastx |
| group_292 | 1130 | Phage | Hypothetical protein [*Pseudomonas syringae*] | Blastx |
| group_995 | 170 | Bacterial | Hypothetical protein [*Pseudomonas syringae*] | Blastx |
| group_920 | 251 | Bacterial | Hypothetical protein [*Pseudomonas syringae*] | Blastx |
| group_926 | 242 | Bacterial | TetR family transcriptional regulator | Bakta |
| group_838 | 359 | Bacterial | Hypothetical protein [*Pseudomonas syringae*] | Blastx |
| group_894 | 293 | Phage | Regulator [*Pseudomonas* sp. JUb42] | Blastx |
| group_1008 | 131 | Bacterial | Division mal foutue 1 protein [*Schizosaccharomyces pombe* strain 972 / ATCC 24843] | HHpred |
| group_951 | 224 | Bacterial | Hypothetical protein [*Pseudomonas syringae*] | Blastx |
| group_1009 | 128 | Not annotated by PHASTEST | Hypothetical protein [*Pseudomonas syringae*] | Blastx |
| group_956 | 218 | Phage | Coiled-coil Trimer with Ala:Leu:Ala Triad | HHpred |
| group_855 | 341 | Bacterial | Hypothetical protein [Multispecies: unclassified *Pseudomonas*] | Blastx |
| group_524 | 815 | Phage | XRE family transcriptional regulator [*Pseudomonas syringae*] | Blastx |
| group_973 | 197 | Phage | Cro/CI family transcriptional regulator [Multispecies: *Pseudomonas syringae* group] | Blastx |
| group_535 | 794 | Phage | KilA-N domain-containing protein [*Pseudomonas syringae*] | Blastx |
| group_905 | 278 | Bacterial | Hypothetical protein [*Pseudomonas syringae*] | Blastx |
| group_536 | 794 | Phage | Pyocin large subunit [Phage] | PHASTEST |
| group_750 | 470 | Bacterial | Protein ninB | HHpred |
| group_608 | 683 | Phage | AlpA family transcriptional regulator | HHpred |
| group_916 | 254 | Phage | Hypothetical protein [*Pseudomonas syringae*] | Blastx |
| group_716 | 518 | Phage | Terminase small subunit [Phage] | PHASTEST |
| group_134 | 1487 | Phage | Terminase [*Pseudomonas syringae*] | Blastx |
| group_743 | 488 | Bacterial | Hypothetical protein KIH13_12515 [*Pseudomonas viridiflava*] | Blastx |
| group_942 | 227 | Bacterial | Mu-like prophage FluMu protein gp35 | HHpred |
| group_840 | 359 | Bacterial | Hypothetical protein [*Pseudomonas syringae*] | Blastx |
| group_1050 | 860 | Phage | Phage tail protein [*Pseudomonas syringae*] | Blastx |
| group_867 | 329 | Phage | Hypothetical protein [*Pseudomonas syringae*] | Blastx |
| group_1054 | 368 | Phage | Phage tail protein [*Pseudomonas syringae*] | Blastx |
| group_708 | 533 | Phage | Glycoside hydrolase family 19 protein [*Pseudomonas syringae*] | Blastx |
| group_721 | 512 | Phage | DUF2514 domain-containing protein [*Pseudomonas syringae*] | Blastx |
| group_799 | 404 | Not annotated by PHASTEST | HNH endonuclease [*Pseudomonas syringae*] | Blastx |
